# The genomic landscape shaped by selection on transposable elements across 18 mouse strains

**DOI:** 10.1186/gb-2012-13-6-r45

**Published:** 2012-06-15

**Authors:** Christoffer Nellåker, Thomas M Keane, Binnaz Yalcin, Kim Wong, Avigail Agam, T Grant Belgard, Jonathan Flint, David J Adams, Wayne N Frankel, Chris P Ponting

**Affiliations:** 1 MRC Functional Genomics Unit, Department of Physiology, Anatomy and Genetics, University of Oxford, South Parks Road, Oxford, OX1 3PT, UK; 2 The Wellcome Trust Sanger Institute, Wellcome Trust Genome Campus, Hinxton, Cambridge, CB10 1HH, UK; 3 Wellcome Trust Centre for Human Genetics, University of Oxford, Roosevelt Drive, Oxford, OX3 7BN, UK; 4 University of California, Los Angeles, California, 90095, USA; 5 National Human Genome Research Institute, National Institutes of Health, Bethesda, Maryland 20892, USA; 6 The Jackson Laboratory, Bar Harbor, Maine 04609, USA

## Abstract

**Background:**

Transposable element (TE)-derived sequence dominates the landscape of mammalian genomes and can modulate gene function by dysregulating transcription and translation. Our current knowledge of TEs in laboratory mouse strains is limited primarily to those present in the C57BL/6J reference genome, with most mouse TEs being drawn from three distinct classes, namely short interspersed nuclear elements (SINEs), long interspersed nuclear elements (LINEs) and the endogenous retrovirus (ERV) superfamily. Despite their high prevalence, the different genomic and gene properties controlling whether TEs are preferentially purged from, or are retained by, genetic drift or positive selection in mammalian genomes remain poorly defined.

**Results:**

Using whole genome sequencing data from 13 classical laboratory and 4 wild-derived mouse inbred strains, we developed a comprehensive catalogue of 103,798 polymorphic TE variants. We employ this extensive data set to characterize TE variants across the *Mus *lineage, and to infer neutral and selective processes that have acted over 2 million years. Our results indicate that the majority of TE variants are introduced though the male germline and that only a minority of TE variants exert detectable changes in gene expression. However, among genes with differential expression across the strains there are twice as many TE variants identified as being putative causal variants as expected.

**Conclusions:**

Most TE variants that cause gene expression changes appear to be purged rapidly by purifying selection. Our findings demonstrate that past TE insertions have often been highly deleterious, and help to prioritize TE variants according to their likely contribution to gene expression or phenotype variation.

## Background

Transposable elements (TEs) have been highly influential in shaping the structure and evolution of mammalian genomes, as exemplified by TE-derived sequence contributing between 38 and 69% of genomic sequence [1-8]. TE insertions also can influence the transcription, translation or function of genes [1-7]. Functional effects of TE insertions include their regulation of transcription by acting as alternative promoters or as enhancer elements and via the generation of antisense transcripts, or of transcriptional silencers. TEs can alter splice sites or RNA editing, provide alternative poly-adenylation signals or exons, modify chromatin structure or alter translation. Furthermore, TE insertion has been suggested to be a mechanism by which new co-regulatory networks arise [1-7].

TEs are classified on the basis of their transposition mechanism [9]. A class I retrotransposon propagates in the host genome through an intermediate RNA step, requiring a reverse transcriptase to revert it to DNA before insertion into the genome. Class II DNA transposons do not have an RNA intermediate, and translocate with the aid of transposases and DNA polymerase. The overwhelming majority, over 96%, of TEs in the mouse genome are of the retrotransposon type [10]. These are further classified into three distinct classes: short interspersed nuclear elements (SINEs), long interspersed nuclear elements (LINEs) and the endogenous retrovirus (ERV) superfamily. The ERVs are ancient remnants of exogenous virus infections, consisting of internal sequence that encodes viral genes that are flanked by long terminal repeats (LTRs) [11].

TEs provide a potential source of variants that are detrimental to host viability and that promote disease. For example, an allele of the *agouti *locus in mouse contains an intra-cisternal A particle (IAP) retrotransposon upstream of the promoter that causes ectopic expression of the agouti protein leading to variation in fur color, obesity, diabetes and tumor susceptibility [12-14]. The murine leukemia virus (MuLV)-like family of ERVs is a potent source of mutagenesis. The first MuLV element insertion was observed to result in hairless and pleiotropic phenotypes [15,16]. That TEs are frequently detrimental can also be inferred from the strong bias in orientation of TEs found in introns of genes. Human intronic ERVs and LINEs, but not SINEs, show a tendency to disrupt expression when inserted into introns in the gene's transcriptional sense orientation [17-21]. In mice there are over 50 examples of phenotypes attributed to spontaneous insertional mutagenesis by ERVs, with one class of functional variants, early transposon (ETn), showing a strong bias to be in the sense transcriptional orientation [6]. This orientation bias is attributed to cryptic splice acceptor usage and/or inefficient read-through of the ERV LTR, which contains its own regulatory signals [6].

TEs that are present in the C57BL/6J reference genome assembly exhibit this orientation bias [17,19-22], which indicates that TE insertions have often been deleterious over tens of millions of years of rodent evolution. By contrast, our knowledge of the intronic distribution and the structure of TE variants (TEVs) inserted during the recent *Mus *lineage has been largely derived from targeted approaches. Previous studies examined two ERV families in eight strains (IAP or ETn/MusD elements in C57BL/6J, A/J, DBA/2J, SPRET/EiJ, CAST/EiJ, MOLF/EiJ, WSB/EiJ and 129X1/SvJ) [18,21,23], with one study in particular focusing on intronic insertions [22] and another exploring LINE variation in four strains (129S1, 129X1, A/J and DBA/2J) [24]. Such TEVs may exhibit a reduced orientation bias because weakly detrimental TEVs that have been inserted, in the sense orientation, during recent evolution may not have had sufficient numbers of generations to be effectively purged from the population. In addition, deleterious TEVs present in laboratory mice might have been maintained owing to their artificial inbreeding. It is thus plausible that TEVs contribute substantially to the genetic load and gene expression variation among inbred and wild mice.

We previously reported the generation and analysis of over a terabase of raw sequence from the genomes of 17 mouse strains [25], and the structural variations called between these strains [26]. In this study we present extensive analyses of a set of TEVs that were subsequently derived from these sequence reads using an updated pipeline that was specifically designed to counter the difficulties inherent in identifying transposition events. We also present this new genome-wide catalogue of ERV, LINE, and SINE TEVs with deep TE subfamily, structural and orientation classifications across 18 (17 newly sequenced plus 1 reference) mouse strains.

This mouse genome project examined 13 classical laboratory (129P2/OlaHsd, 129S1/SvImJ, 129S5/SvEvBrd, A/J, AKR/J, BALB/cJ, C3H/HeJ, C57BL/6NJ, CBA/J, DBA/2J, LP/J, NOD/ShiLtJ and NZO/HiLtJ) and 4 wild-derived mouse inbred strains (CAST/EiJ, PWK/PhJ, WSB/EiJ and SPRET/EiJ), and each was compared to the C57BL/6J reference sequence. Altogether, this group encompasses approximately 2 million years (My) of evolutionary divergence [27]. Concomitantly, RNA-Seq data were generated from whole brain tissue from 14 of the 17 mouse strains, thereby allowing us to consider the impact of genotypic differences on gene expression levels.

By taking advantage of these new data we provide a comprehensive analysis of the patterns of variation of all three TE classes across the *Mus *genus, and use these data to examine the extent to which genomic location influences the retention or purifying selection of TEVs among a large number of mouse strains. We first show variable ERV, LINE and SINE variant densities across the 18 mouse genomes, within chromosomal, intergenic and genic locations, and for different gene classes, and then account for these variable densities by invoking processes of neutral evolution and purifying or positive selection.

## Results

### Genome landscape of recently inserted TEVs

**Figure 1:**
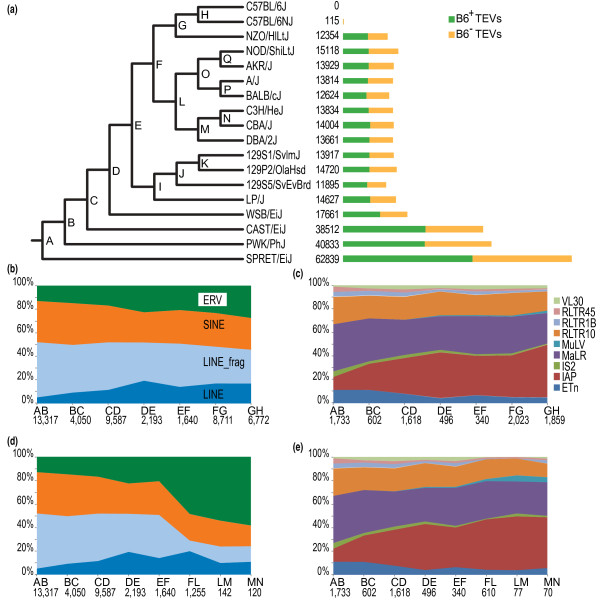
**Distribution of TEVs across a phylogeny representing a primary subspecies history of 18 mouse strains**. **(a) **This phylogeny (left), which averages across these strains' known phylogenetic discordances, was imputed by considering TEVs to be discrete morphologies. All nodes were supported by bootstrap values of 100%. Numbers of B6^+ ^and B6^- ^TEV insertions are shown (right), within and without the C57BL/6J lineage, respectively. Unless we have evidence for the contrary, we assume that a strain's genome is identical to that of C57BL/6J. Green and yellow bars represent numbers of TEV insertions in the C57BL/6J lineage (B6^+^; green) and insertions outside of this lineage (B6^-^; yellow), respectively. The large number of TEVs in SPRET/EiJ (bottom) indicates that these are most often absent in C57BL/6J (and other lab-based strains), rather than indicating that there is a larger number of TEs in SPRET/EiJ. **(b, d) **Proportions of TEV classes (ERVs, LINEs and SINEs) across the inferred phylogeny for C57BL/6J (b) and C3H/HeJ (d) lineages. LINE elements are further divided into full-length insertions (> 5 kb) and smaller fragments of LINEs (LINE_frag). **(c, e) **Proportions of ERV families across the inferred phylogeny for C57BL/6J or C57BL/6NJ (c) and C3H/HeJ or CBA/J (e) lineages. For example, 'AB' indicates insertion events inferred to have occurred after the divergence of SPRET/EiJ from all other strains. Numbers of predicted insertions are given below.

We computationally predicted 103,798 TEVs (28,951 SINEs, 40,074 LINEs and 34,773 ERVs) among the 17 sequenced mouse strains in addition to the C57BL/6J reference strain; 6 of these have previously been examined in some respects (129S1/SvImJ, A/J, DBA/2J, CAST/EiJ, WSB/EiJ and SPRET/EiJ), whereas others have, to our knowledge, not been systematically examined for TEVs (129P2/OlaHsd, 129S5/SvEvBrd, AKR/J, BALB/cJ, C3H/HeJ, C57BL/6NJ, CBA/J, LP/J, NOD/ShiLtJ, NZO/HiLtJ and PWK/PhJ) (Additional file 1). This number of TEVs is over four times higher than for all previous studies combined [18,21,23,24]. We employed two approaches for TEV discovery, SVMerge, which combines the results of four methods of structural variant prediction [28], and RetroSeq (Additional file 2). After filtering (Materials and methods), SVMerge predicted 44,401 non-redundant insertions within the lineage of the C57BL/6J reference strain, whereas the RetroSeq method inferred 59,397 TEV insertions occurring outside of this lineage (Figure 1a). We refer to these insertions, in the reference strain lineage and outside the reference lineage, as B6^+ ^and B6^- ^TEVs, respectively (B6^+^, TE present in C57BL/6J; B6^-^, not present in C57BL/6J). By further classifying TEVs according to type and class, we determined that virtually all mouse strain TEVs are drawn from subfamilies that were previously observed to be active [9] (Figure 1; Additional file 3). Each strain has approximately equal numbers of B6^+ ^and B6^- ^variants, as one might expect if TEVs accumulated at similar rates. There are higher numbers of TEVs relative to C57BL/6J in the wild-derived strains (SPRET/EiJ, PWK/PhJ and CAST/EiJ; 13.8 to 22.4 per Mb) than in the laboratory strains (4.2 to 6.3 per Mb; Figure 1a). By examining the strain distribution patterns derived from 688 PCR validation of TEVs across 8 strains, we find relatively low false positive rates (11 to 22%); furthermore, by conservatively assuming the genomes of the three 129-derived substrains (129S1/SvImJ, 129P2/OlaHsd and 129S5/SvEvBrd) to be identical, we estimated false negative rates to be 5 to 28% across the classes (Additional files 4, 5 and 6). Given the available read length, insert size and coverage, there were limits to the degree to which subfamilies of TEVs could be reliably stratified. Differences in our ability to distinguish TEV subfamilies are attributable to contrasting sequence divergence in their first and last 300 bp. SINE subfamilies could not be distinguished, LINEs were classified as either full-length LINEs or LINE fragments, and ERVs were placed into subfamilies VL30, RLTR45, RLTR1B, RLTR10, MuLV, MaLR, IS2, IAP and ETn. However, the range of classes and families we investigated, to our knowledge, represents the broadest study of TEVs in the *Mus *lineage to date.

In order to interpret this vast catalog, we placed the TE insertions within a primary phylogeny of these mouse strains, which permitted an initial overview of the relative expansions of all the TE families over an approximate 2 My time period (Figure 1a). This primary phylogeny matched the phylogeny expected from the heritage of the mouse strains [29]. This analysis revealed the historic expansion of ERV families, most notably IAPs, in laboratory strains (Figure 1b-e). ERVs were seen to contribute between 29 and 39% of all TEVs in the sequenced strains (Figure 1b-e).

**Figure 2:**
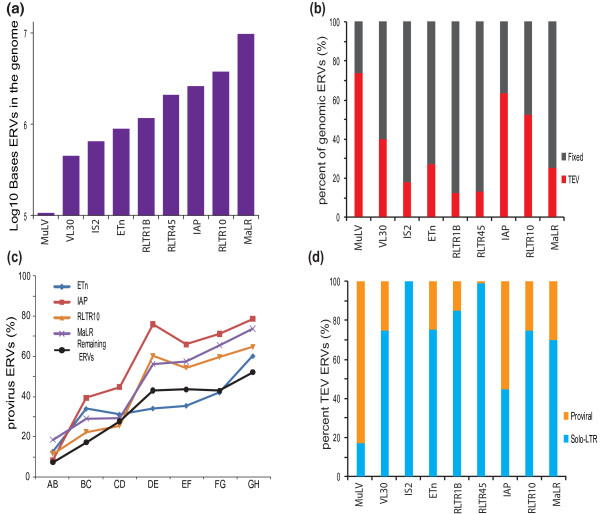
**Structure and activities of ERV families**. **(a) **Numbers of bases within nine ERV families present in the C57BL/6J reference genome. **(b) **Proportions of C57BL/6J bases belonging to each ERV TEV family relative to apparently fixed ERV sequence. These proportions reflect the variable ages and historical activities of ERV families. **(c) **Percentages of TEV ERVs predicted as having a proviral structure within the C57BL/6J or C57BL/6NJ lineages projected onto the phylogenetic tree from Figure 1. An older ERV insertion shows a greater tendency to be a solo-LTR than a canonical proviral form. **(d) **Proportions of proviral and solo-LTR structures for each TEV ERV family. Solo-LTR fractions are approximately proportional to the age of the TEVs with the exception of ETn and IAP, which have a higher percentage of solo-LTRs for their age relative to the other families.

Different ERV families contribute, in sequence length, to greatly differing extents to the mouse genome (Figure 2a). The relative proportions of polymorphic, and apparently fixed ERVs, also vary considerably among these families (Figure 2b), in part reflecting the ages of past exogenous viral infections. The MuLV family, for example, arose recently and thus is found in a smaller number of copies that together show a higher fraction of variable elements (Figure 2a, b). ERVs are prone to recombination between their flanking LTR sequences. To estimate this recombination rate in different ERV families, we mapped TEVs to the inferred mouse strain primary phylogeny and observed increasing proportions of solo LTR elements with increasing phylogenetic divergence (Figure 2c). We estimate the average half-life for ERV recombination from provirus to solo-LTR to be approximately 0.8 My (assuming a constant rate and that all ERVs insert as proviruses; Figure 2c, d; Materials and methods). IAP elements recombine particularly rapidly, with a half-life we estimate to be approximately 0.7 My years (Figure 2c, d; Materials and methods). This is similar to a previous estimate derived from a single MuLV element within the *dilute *locus of DBA/2J mice [30]. By contrast, the older family of ETn elements appear to be associated with a much slower rate of recombination. The differences in recombination rate may, in part, reflect variations in LTR lengths among different ERV families.

**Figure 3:**
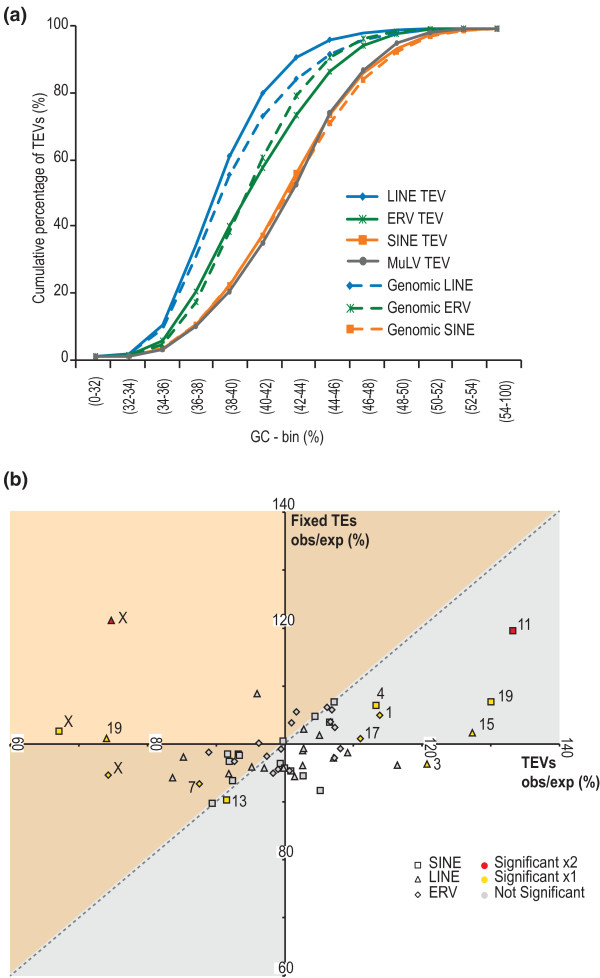
**Genome-wide nucleotide composition and chromosome biases for TEV density**. **(a) **Cumulative distributions of TEV families according to their genomic GC context. SINE TEVs tend to occur in GC-rich sequence while LINE and ERV TEVs each show an AT preference, with the notable exception of the MuLV family, which is biased towards GC. TEVs showed no differences in these biases compared to all TEs in the reference genome assembly. **(b) **Chromosome biases in the densities of TEVs (x-axis) and apparently fixed TEs (y-axis). Each axis represents the observed density divided by the density expected from genome-wide random samples of sequence approximately matched according to G+C content (Materials and methods). Significant density deviations from the null expectation are indicated by color for TEVs or fixed TEs (yellow) or both (red). The spread in observed densities across chromosomes is greater for TEVs compared with the older, apparently fixed, TEs. There is a general tendency for chromosomes that exhibit elevated (decreased) TE densities to also exhibit increased (lower) densities of TEVs (excluding chromosome X: SINE R^2 ^= 0.6756, *P *< 10^-4^; LINE R^2 ^= 0.0054, *P *< 0.7; ERV R^2 ^= 0.3836, *P *< 0.0047). The quadrants shaded grey match this correlation in ratios between TEVs and fixed TEs. Nevertheless, this trend does not explain the higher than expected density of TEVs on the X chromosome when compared with its lower than expected TE density. All chromosomal TE density points that fall within the orange shaded area show signals of positive selection according to the McDonald-Kreitman test (FDR 0.1%).

TEV density varies by chromosome, by local nucleotide composition (G+C content) [31-33], and by position relative to functional sequence, such as exons. LINE TEVs show a bias for being located in A+T-rich sequence, whilst SINE TEVs tend to reside in G+C-rich sequence (Figure 3a) [34,35]. We also observed ERV TEVs to be more heterogeneous than SINEs or LINEs in their G+C bias, with MuLV TEVs being as enriched in high G+C sequence as SINEs (Figure 3a). In subsequent analyses we determined the extent and significance of enrichments and depletions by implementing a genome-wide association procedure that accounted for three potentially confounding effects, namely the different rates of TE insertion across (a) the G+C content spectrum (Figure 3a), (b) different chromosomes (Figure 3b), and (c) sequence of varying length (Materials and methods) [36].

We find that apparently fixed TEs (SINEs, LINEs or ERVs) occur at relatively even densities across individual chromosomes having accounted for G+C content (Figure 3b, y-axis). An exception to this are SINEs on chromosome 11, which is unusual in at least two respects, namely its elevated replication rate [37] and gene density. Nevertheless, when we took account of these factors in this analysis, the strong enrichment of SINEs remained essentially unchanged (data not shown; Materials and methods). The second notable exception are LINEs on the X chromosome [34,35], whose higher density has been attributed to increased rates of LINE insertions in both male and female germlines [38].

Interestingly, by contrast to monomorphic TEs, polymorphic TEVs are more unevenly distributed among the chromosomes (having accounted for G+C content) with, for example, chromosome 19 exhibiting a significant surfeit of SINEs and the X chromosome showing a strong deficit of all three TEV classes (Figure 3b, x-axis). The depletion of polymorphic LINEs on the X chromosome was previously seen in a study of four mouse strains (A/J, DBA/2J, 129S1/SvImJ and 129X1/SvJ) [24]. These G+C-accounted TEV biases on autosomes (A) and on the X-chromosome allowed us to calculate the male TEV insertion bias: α = (3(X/A) - 4)/(2 - 3(X/A)) [39]. We obtain α values of 7.8 (95% confidence interval (CI) 4.7 to 13.2), 7.3 (95% CI 5.5 to 11.9) and 151.8 (95% CI 18.5 to ∞) for ERV, LINE and SINE TEVs, respectively. These estimates of male TEV insertion bias are 3- to 68-fold higher than estimates based on substitution rates [40] and imply that TE insertions occur almost exclusively in the male germline genome. By adapting the McDonald-Kreitman test [41] (Materials and methods), we considered whether the ratio of fixed to polymorphic TEs is indicative of the past action of positive selection on TEVs (Figure 3b). Our results corroborate previous proposals of positive selection on LINE TEVs on the X chromosome [42,43] (*P *< 10^-16^). Our study, however, has the advantage of using genome-wide observations of fixed and polymorphic TEs. This approach also predicts, for the first time, positive selection for preferential retention of ERV and SINE TEVs on the X chromosome.

### Purifying selection on TE insertions within genes

**Figure 4:**
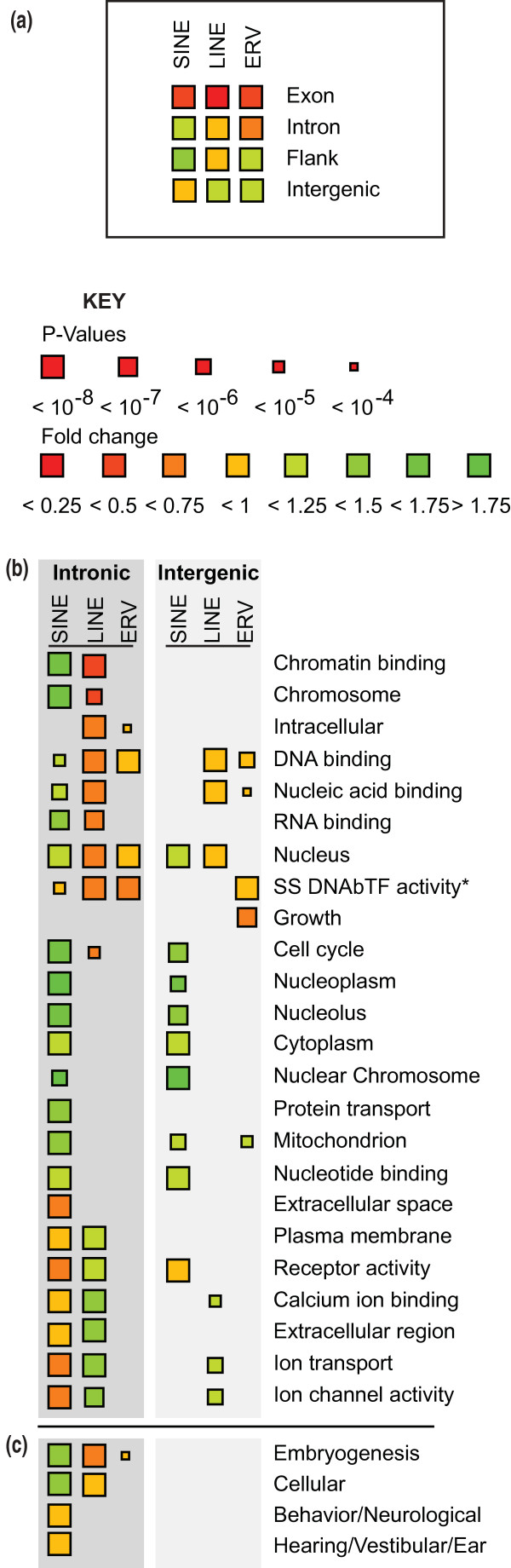
**Genome-wide nucleotide composition, gene structure and gene annotation biases for TEV occurrence**. **(a) **Having accounted for these GC biases, TEVs are substantially and significantly depleted (red shades) in exons and introns, with the notable exception of SINEs that are enriched (green shades) in intronic regions. Both SINE and ERV TEVs are enriched in 5 kb upstream and downstream flanking regions of genes, while LINEs are depleted. **(b, c) **Gene annotations that are significantly enriched (green shades) or depleted (red shades) in intronic or intergenic TEV insertions having accounted for GC content, and intronic or intergenic lengths, and after adjusting for multiple tests. Gene annotations are from either the Gene Ontology (slim set) (b) or the Mouse Genome Informatics phenotypes associated with gene disruptions (c), and are shown when at least one significant association (*P *< 10^-6^) was observed. SINE TEVs show a pattern of enrichments and depletions that is the complement of the patterns for LINE and ERV TEVs. * Sequence-Specific DNA binding Transcription Factor activity.

TEVs from all three classes show strong and significant depletions in protein-coding gene exons (Figure 4a), implying that such insertions are strongly deleterious (assuming that most TEVs across the noncoding genome are neutral or deleterious). Using our genome-wide association procedure, we tested for the over- or under-representation of TEVs across the genome within introns, or 5 kb flanking sequences of protein coding genes, or within the remaining intergenic sequence. No significant differences were found in the densities of SINE, LINE or ERV TEVs between first, middle or last introns (data not shown). However, SINE TEVs were enriched in flanking and intronic sequence, in contrast to LINE and ERV TEVs, which were strongly depleted in introns (false discovery rate (FDR) < 0.1%; Figure 4a).

We then considered whether intronic TEV densities are higher in genes from particular functional classes (Gene Ontology (GO) and Mouse Genome Informatics annotations; Materials and methods), once again accounting for chromosome and nucleotide composition biases. The introns of genes with essential housekeeping functions, such as transcription and chromatin binding factors, and genes that are associated with embryogenesis phenotypes, were observed to be significantly and strongly depleted in LINE and ERV TEVs (Figure 4a, b; Additional file 7). In contrast, housekeeping genes show a significant enrichment of intronic SINE TEVs (Figure 4b).

Next, we calculated the orientation bias (Orientation bias = (TEVs in sense orientation)/(All TEVs)) for the 20,001 intronic TEVs for which we had orientation data. If TE insertions are random, and are not frequently deleterious, or if they are only mildly deleterious, then we would expect to observe no bias (orientation bias ≈ 50%). Instead, a strong orientation bias was evident for each of the three TE classes (32.6%, 41.7%, and 41.6% for ERV, LINE and SINE TEVs, respectively). The orientation bias for IAP TEVs was recently reported to be 25.9% for a redundant set of 3,317 intronic IAPs [18]. This is lower than our non-redundant set of 2,418 intronic IAP TEVs (orientation bias = 30.9%). The strong biases for ERVs and, to a lesser extent for LINEs, are consistent with these elements being depleted from introns (Figure 4a). The orientation bias for SINE TEVs indicates that despite their enrichment in introns (Figure 4a), which is assumed to reflect a mutational bias, they are strongly depleted when inserted in the transcriptional sense orientation. TEV orientation biases were no different for genes annotated with GO terms found either to be enriched or to be depleted in TEVs (*P *> 0.05, χ^2 ^test; Figure 4b).

**Figure 5:**
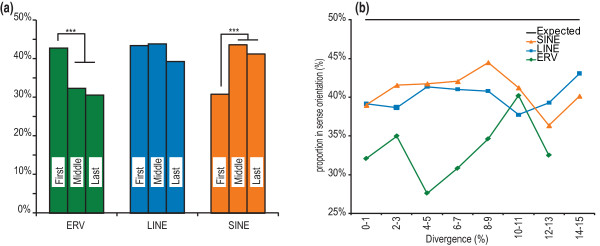
**Densities and orientations of TEVs with respect to the transcriptional (sense) direction of mouse genes**. **(a) **Orientation bias within first, middle and last introns of protein coding genes. All TEV types occur preferentially in the antisense orientation, with the ERV TEV bias being the strongest. ERV TEVs show a lower bias in the first introns of genes (*P *< 10^-3 ^by chi-square test). SINE TEVs show a significantly stronger orientation bias in the first introns of protein coding genes (*P *< 10^-3^). **(b) **Orientation biases are not significantly different between 'young' and 'old' TEVs, categorized using percentage sequence divergence from the repeat consensus sequence (x-axis). *** indicates *P *< 10^-3^.

The large set of TEVs in this study allowed us to infer whether the location of a TEV within a gene structure affects the strength by which it is purified from the population. Orientation bias was significantly stronger for ERV TEVs within middle or last introns, and for SINE TEVs within first introns (Figure 5a). We find the orientation bias not to be significantly different between genes with high or low brain expression (data not shown) or between TEV classes that are relatively young (little divergence) or old (Figure 5b), or between solo-LTRs and proviral LTRs (data not shown).

By comparing the orientation bias for TEVs, which were inserted relatively recently, with the corresponding bias for predominantly older monomorphic TEs, we were able to infer the rate by which each TEV class is purged. Orientation bias was not significantly different between apparently fixed and variant ERV TEs (Table 1). By contrast, orientation bias was significantly and substantially stronger for apparently fixed LINE TEs than for recently inserted LINE TEVs. There was also a small, yet significant, increase in the strength of this bias for SINE TEVs relative to apparently fixed SINE TEs.

**Table 1:** Orientation bias values of TEVs or apparently fixed TEs in the mouse genome

	Sense	Antisense	Percentage sense	Chi-square test *P*-value
ERV TEV	2,042	4,176	32.8%	0.61
Apparently fixed ERV	61,857	128,263	32.5%	
LINE TEV	3,803	5,336	41.6%	3. 5 × 10^-33^
Apparently fixed LINE	81,655	148,497	35.5%	
SINE TEV	4,192	5,528	43.1%	1.2 × 0^-4^
Apparently fixed SINE	282,011	343,528	45.1%	

For each TE superfamily the number of intronic variants within mouse genes with human orthologs was counted. B6^+ ^TEVs and apparently fixed C57BL6/J TEs were counted as either sense or antisense with respect to the gene's transcriptional orientation. Significant differences between the orientation biases of apparently fixed and variant TEs were obtained using a chi-square test.

### Purifying selection on TE insertion depends on proximity to functional elements

**Figure 6:**
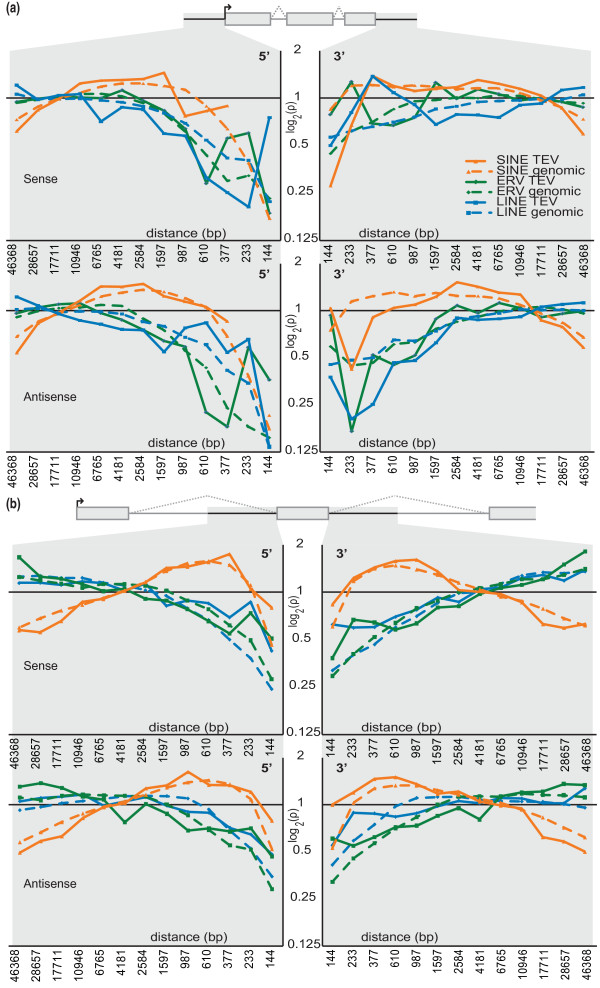
**Densities of intergenic TEVs in the proximity of gene boundaries**. **(a) **Densities of TEVs (full lines) or of TEs from the reference C57BL/6J assembly (dashed lines) 5' of genes' transcriptional start sites (left panels) or 3' of genes' transcriptional stop sites (right panels). The top two panels represent TEVs and TEs that occur in the transcriptional sense orientation, whereas the bottom two panels represent those present in the antisense orientation. For each family, the densities of TEVs (y-axis) present within distance bins (x-axis) from the gene are shown relative to the TEV density observed. Bin sizes were selected from the Fibonacci series, which allowed improved visualization of TEV densities compared to linear or logarithmic scales. All TEVs and TEs are depleted in close proximity to the 5' of genes, but SINEs are enriched upstream (approximately 500 bp to 10 kbp) of genes. No significant effects of TEV orientation on density distributions in the vicinity of genes were observed. **(b) **Densities of intronic TEVs in the proximity of exon boundaries. Densities of intronic TEVs (full lines) or of TEs from the reference C57BL/6J assembly (dashed lines) 5' of exons (left panels) or 3' of exons (right panels). The top two panels represent TEVs and TEs that occur in the transcriptional sense orientation, whereas the bottom two panels represent those present in the antisense orientation. For each family, the densities of TEVs (y-axis) present within distance bins (x-axis) from the gene are shown relative to the TEV density observed. Bin sizes were selected from the Fibonacci series, which allowed improved visualization of TEV densities compared to linear or logarithmic scales. A difference in density profiles of sense and antisense TEVs is observed in proximity to exon boundaries.

Strong purifying selection of TEVs from all three classes, and in both transcriptional orientations, is evident in sequence near (< 0.5 kb) to the transcriptional start sites of genes (Figure 6a). Purifying selection of deleterious TEVs appears less strong near the 3' of genes. We observed a significant increase in SINE TEVs in the vicinity of genes (as previously observed [19]) and upstream and downstream (1 to 10 kb) of genes (*P *< 10^-16^; Figure 6a).

A recent study of 161 mouse ERV TEVs identified their strongest intronic orientation bias to be in the close vicinity of exon boundaries [22]. Using our larger set of 20,001 intronic TEVs, we confirmed this finding, and then extended it to include all TEVs (Figure 6b). SINE TEVs exhibit a reduced orientation bias near exons, thus appearing to be less deleterious; their depletion within the interiors of introns appears to reflect a G+C composition bias [22].

### Impact of TEVs on quantitative traits and expression levels

**Figure 7:**
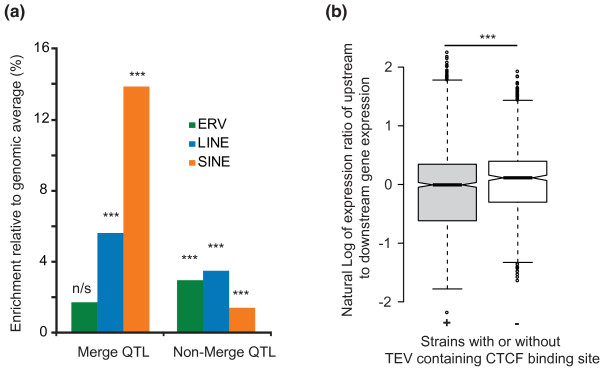
**Enrichment of TEVs within merge QTL regions and functional impact of CTCF-binding TEVs**. **(a) **The density of TEVs within genomic intervals associated with refined ('Merge') QTLs or adjacent sequence lying within nonrefined QTLs relative to all genomic regions was tested using a genome-wide association test. SINE TEVs show a 13.9% enrichment in regions associated with refined merge QTLs over the level expected given interval size and GC composition. Both LINE and SINE TEVs show greater densities within merge QTL regions compared to surrounding non-merge QTL sequence. With the exception of ERV TEVs in merge QTL regions, all associations were statistically significant (***P < 10^-3^). **(b) **Variation in expression between genes associated with a CTCF-binding TEV. The natural log of the ratio of expression between the upstream and downstream genes (whose order was randomly assigned) was taken as a measure of the variance in expression. The presence of the CTCF binding TEV was associated with a greater degree of expression variation between flanking genes (ANOVA *P *< 0.001). N/s, not significant.

To consider whether evidence could be obtained that TEVs commonly contribute to mouse quantitative traits, we used genomic loci (quantitative trait loci (QTLs)) that have been further refined using a merge analysis [25,44]. Using our genome-wide association procedure, we found small but significant enrichments of LINE and SINE TEVs within these refined genomic intervals (Figure 7a, Merge QTLs). By contrast, these enrichments were reduced in sequence lying outside of these refined intervals but remaining within their overarching QTLs (Figure 7a, Non-merge QTLs). These results provide evidence that TEVs contribute, to a limited extent, to at least some of the approximately 100 quantitative traits under consideration. Of the 12 variants that passed the genome-wide association with QTLs [26], two were found to be IAP TEVs.

To establish a set of TEVs that likely affect gene expression, we examined the dataset for previously proven examples of ERV strain variants, of which there are about a dozen examples [6] (also see [45]), of which only some would show sufficient expression in brain to be detected in our study. Indeed, for ERV insertions in *Gria4 *(IAP type I insertion specific to C3H/HeJ mice), *Myo5a *(MuLV insertion specific to DBA/2J) and *Zhx2 *(ETn insertion specific to BALB/c), the expected expression decreases associated with the ERV allele were all observed (data not shown). However, since many ERV insertion effects are often local, primarily affecting levels of a nearby exon [45], and/or are cell type-specific [46], these and likely other novel TEV *cis *effects on gene expression did not survive the stringent multiple testing correction required for genome-wide analysis.

We next considered whether TEVs, when considered together, were often causal variants of gene expression changes or quantitative traits. We compared expression levels among strains, acquired from RNA-Seq experiments of whole brain samples, for genes with or without an intronic TEV. Ascribing a gene expression difference to a specific TEV is confounded by the presence of other linked variants that may, instead, be causal. To account for this and to identify a conservative set of TEVs associated with gene expression differences, we calculated in each strain the expression for the constitutively expressed sequence and then normalized these mapped read levels between samples to allow for their comparison. By considering the presence or absence of a TEV as an experimental condition, differential expression of genes was calculated (Materials and methods). In a previous publication [26], we estimated that the proportion of expression heritability attributable to TEVs is no more than 10% across all genes. Despite this, we identified 28 of 48 differentially expressed genes having one or more TEVs (Additional file 8). TEVs were thus found two-fold more frequently associated with differential expression of genes into which they have been inserted than expected by chance (*P *< 0.01). We found no significant bias for the direction of expression change between strains associated with these TEVs. This finding then allowed us to investigate whether a specific TEV class contributes greatly to these gene expression differences. We would expect that when we account for the different TEV densities in genes, ERV, LINEs and SINEs would be equally likely to be associated with differential gene expression. However, we found ten-fold fewer LINEs and LINE fragments associated with differential gene expression than expected (*P *< 0.01). This implies that LINE insertions that cause changes in gene expression are substantially more likely to be purged by purifying selection than are ERV and SINE TEVs.

**Figure 8:**
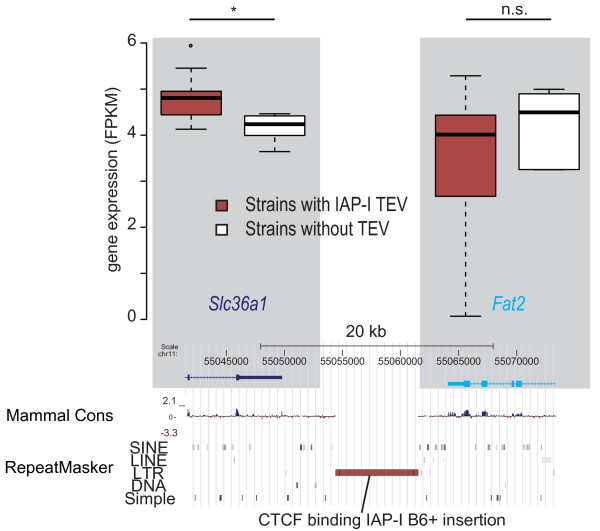
**An example of a genomic region with a CTCF-binding TEV, in this case an IAP-I provirus**. The presence of the IAP-I is associated with differential expression of *Slc36a1 *(*P *< 0.05, genome wide FDR 0.05) across the strains but not of *Fat2*. The IAP-I is present in the strains 129P2/OlaHsd, 129S1/SvImJ, 129S5/SvEvBrd, A/J, AKR/J, BALB/cJ, C3H/HeJ, C57BL/6NJ, CBA/J, DBA/2J, LP/J, NOD/ShiLtJ, CAST/EiJ, PWK/PhJ and WSB/EiJ.

A recent publication showed that species-specific CTCF binding sites frequently occur in TEs [47]. To examine if variation in gene expression could be associated with the presence or absence of such CTCF-associated TEVs, we intersected the B6^+ ^TEVs with these CTCF binding site region predictions. We observed an increased expression variation between genes that flank a CTCF-binding TEV. The likelihood for this change was smaller than that obtained for the expression variance increase for 10,000 random samples (that is, empirical *P *< 10^-4^; Figure 7b). Thus, the increase in expression variation is associated with CTCF-binding TEVs specifically, rather than with TEVs in general. In Figure 8 we present an example of a CTCF-binding TEV, an IAP-I element lying between *Slc36a1 *and *Fat2 *on chromosome 11 in all but three strains (DBA/2J, NZO/HiLtJ and SPRET/EiJ).

To provide our data to the research community as a resource, we provide in Additional file 9 a table listing 455 intronic TEVs, together with their associated genes' brain RNA expression values, that are significantly associated with expression differences, after controlling for false discoveries (455 TEVs within 322 genes). A similar table (Additional file 10) lists 1,404 intergenic TEVs, and their significant associations with the expression of neighboring genes (1,404 TEVs, neighboring 776 genes).

## Discussion

Deep sequencing of 17 mouse genomes has allowed substantial improvements in the numbers and accuracies of TEV calls. We present a near complete catalogue of 103,798 TEVs that have survived selection and drift over the past 2 My of *Mus *lineage evolution. From an inferred evolutionary history of TE family activity, ERVs and in particular the IAP subfamily, appear to be expanding rapidly in mouse genomes, as previously proposed [6].

### Chromosomal distribution of TEVs

Our findings allow a genome-wide comparison between mostly fixed C57BL/6J TEs and TEs that are segregating among the strains. We found that, similarly to fixed TEs, LINE TEVs show a location preference for A+T-rich sequence, whilst SINE TEVs tend to reside in G+C-rich sequence (Figure 3). As was noted previously [10], these opposing tendencies are perplexing since LINEs and SINEs insert using the same endonuclease. One possible resolution to this puzzle is suggested by 'older' SINEs showing G+C distributions that differ from those for more recently inserted SINEs, perhaps because SINEs in A+T-rich sequence are more readily deleted [48]. If so, then we would expect G+C distributions for recent TEV insertions to differ from those for all TE insertions. Indeed, it has been proposed that observed genomic distributions will differ substantially from the original insertion site preferences, owing to a combination of selection and genetic drift [22]. However, we see no evidence of ongoing selection on SINE G+C bias, implying either that the selection is relatively rapid or that there is an unobserved bias at work.

MuLV TEVs show a higher G+C base composition preference compared to other ERV TEVs. As a consequence, MuLV elements are more likely to be inserted in sequence that is rich in functional elements, and thus may have a greater propensity for modifying gene expression levels. Indeed, MuLV insertions have already been observed to modulate the expression of several proto-oncogenes in tumors [16].

LINE, SINE and ERV TEVs are all depleted on the X chromosome, with observed densities approaching two-thirds of the autosome densities expected from an insertion pattern that is exclusively male (in contrast to equal densities if there are no gender mutation biases). This has previously been observed to be the case for active *Alu *elements in humans [49]. We propose that the vast majority of all TE insertions in the *Mus *lineage have occurred in the male germline genome. While the degree of inter-chromosomal variation is decreased for apparently fixed ERV and SINE TEs in the genome, monomorphic LINE TEs are enriched on the X chromosome. We can conceive no alternative model other than strong positive selection for this apparent higher rate of fixation in excess of the rate of mutation.

### Purifying selection on intronic TEVs

Variation in TEV densities in intergenic and genic regions compared with expected values indicate ongoing selective processes acting on TEVs. The significant deficits of ERV or LINE TEVs in introns indicate that many were deleterious and thus were selectively purged over these strains' evolutionary history. These observations agree with previous findings that LINE TE insertions are less well tolerated within gene-rich sequence [38]. While LINE and ERV TEVs are strongly depleted in genes associated with housekeeping functions, SINEs are enriched in such genes (Figure 4). *De novo *intronic insertion variants of LINEs or ERVs but not SINEs are thus likely to often dysregulate such genes. Interestingly, it is exactly these classes of genes whose regulation depends on TE-derived sequence [50]. The enrichment of SINE TEVs, in contrast to the depletions of ERV and LINE TEVs, is consistent with a previous proposal that SINEs preferentially insert within genes that are expressed in the germ line [51], and is in agreement with the increased density of SINE TEVs in the vicinity of genes [19]. While all TEV classes are depleted in the immediate proximity of genes and splice donor and acceptor sites, SINEs are enriched within 10 kb of genes and have shorter exclusion zones near to functional sequence compared to ERV and LINE TEVs.

If it is assumed that TEVs that are inserted in the antisense orientation are not under selection, then orientation bias values imply that approximately 50% of all ERVs that inserted in the sense orientation into the introns of protein coding genes have been deleterious, as have been about one-third of LINE or SINE sense insertions. We might expect TEV orientation biases to be different between genes annotated with GO terms found either to be enriched or to be depleted in TEVs. However, no such differences were detected, which implies that antisense orientated TEVs may also commonly be deleterious (perhaps by affecting the expression of antisense transcripts), and that the ratio of sense-to-antisense deleterious TEVs is relatively constant among genes from different functional classes.

The observed orientation biases do not appear to be attributable to non-selective mutational or repair mechanisms. This is because we observe differences in orientation bias values for SINE and ERV TEVs depending on intron position in a gene, which in turn make less likely alternative models that explain orientation bias as being due solely to a mutational preference for TE preservation in the antisense strand, perhaps as a consequence of transcription-coupled repair [52].

No substantial differences in orientation bias values between fixed and variant ERVs were observed. This implies that deleterious sense inserted ERV TEVs are not commonly segregating among these mice; rather, they have most often been purged very rapidly from the mouse population. Orientation bias was significantly and substantially stronger for apparently fixed LINE TEs than for recently inserted LINE TEVs, implying once more that purifying selection on sense inserted LINE TEVs tends to be less strong than on ERVs.

### Effect of TEVs on gene expression and quantitative traits

Regions annotated with refined QTLs [44] are significantly enriched in LINE and SINE TEVs. This is evidence that TEVs contribute, albeit rarely, to at least some of the approximately hundred traits that were considered. Many of these effects are likely to act either on exons flanking the TEV site, or on downstream elements, as has been observed for many known *de novo *ERV insertions [6]. Although a small number of TEVs (132) were annotated as being in coding exons, due to breakpoint uncertainty some of these will instead be intronic. None of our ERV, LINE or SINE TEV sets was significantly associated with global expression level change measured using an RNA-Seq experiment of whole brain samples. This suggests that TEVs that survive purifying selection are only rarely associated with gene expression changes. Although *de novo *TE insertions frequently cause disruptions in genes' expression, such deleterious variants appear to be often purged by strong purifying selection and the genomic contribution of the remaining TEs to global gene expression variation thus appears minimal.

Clearly a subset of TEVs will affect the expression of their resident genes and their functions. Indeed, using a stringent statistical re-sampling approach to take into account confounding influences of strain and expression divergence, we found that TEVs are twice as likely to reside in a differentially expressed gene as expected by chance. Thus, among all genes that exhibit expression differences between strains, TEVs contribute more than expected by chance. Only 34 TEVs passed a stringent genome-wide test, and these TEVs contain significantly fewer LINEs than the null expectation that all TEV classes have equal effects. While it has been extensively documented in the literature that *de novo *LINE insertions can cause changes in gene expression, it appears that, in *Mus musculus*, purifying selection has preferentially purged such variants.

However, given that the proportion of expression heritability attributable to TEVs generally is no more than 10% [26], many of the significant expression changes tabulated in Additional files 9 and 10 will not be due specifically to the TEV but rather, for example, to co-segregating variants. Nevertheless, these data, together with evidence that the insertion and selection on TE insertions vary considerably according to class, transcriptional orientation, inter- or intragenic location, and gene functional category should now assist in distinguishing the minority of TEVs with a profound negative effect on organismal fitness from the majority of TEVs with little to no effect on fitness. There are a host of possible phenotypic consequences of TEVs outside the ones tested here, such as premature transcriptional termination at a distance triggered by ERVs [18]. It is also likely that some TEVs have phenotypic effects that are restricted to specific tissues and/or developmental time points. Although determining the full extent of such effects is beyond the remit of this paper, the extensive catalogue of TEVs that we have presented provides a valuable resource that should greatly facilitate such studies.

## Conclusions

We present a near complete catalogue of TE variation across 18 mouse strains, encompassing 2 My of divergence within the *Mus *lineage. We recaptured previously reported variation in the relative activities of different mouse TE families and also report evidence that the vast majority of TE activity has occurred in the paternal germ line. Strong signals of purifying selection are evident with respect to TE family, genomic location, orientation and functional category of encompassing genes. Most TEVs that are not purged by rapid and strong negative selection appear to have little, or no, effect on organismal fitness. Nevertheless, we found that a small fraction of TEVs are associated with relatively large effects on gene expression.

## Materials and methods

### Sequencing data

Raw sequencing data were generated from 13 classical laboratory (129P2/OlaHsd, 129S1/SvImJ, 129S5/SvEvBrd, A/J, AKR/J, BALB/cJ, C3H/HeJ, C57BL/6NJ, CBA/J, DBA/2J, LP/J, NOD/ShiLtJ and NZO/HiLtJ) and 4 wild-derived (CAST/EiJ, PWK/PhJ, WSB/EiJ and SPRET/EiJ) mouse inbred strains as part of the Mouse Genomes Project [25]. Briefly, 1,239 Gb of mapped sequence were generated using the Illumina GAIIx platform [53] providing an average of 27.6-fold sequence coverage across 17 genomes. Paired-end reads were a mixture of 37 bp, 54 bp, 76 bp and 108 bp in length, with fragments being 150 to 600 bp in length. Accession numbers for the raw sequencing data are given in Additional file 1.

### ERV probes

Differences in nomenclature and classification groupings between RepBase/Repeatmasker and colloquial descriptions used in the literature can be difficult to resolve. In this study, we classify TEs into DNA elements, SINEs, LINEs and ERVs. ERVs have been further classified into the following families: IAP, ETn, MuLV, VL30, MaLR, RLTR10, IS2, RLTR45 and RLTR1B. A complete listing of RepBase [54] classifications and conventional ERV super-classes corresponding to these families is provided in Additional file 2.

### B6^+ ^calling algorithms

Structural variant (SV) deletions in all inbred strains were detected using three methods: split-read mapping (Pindel [55]), mate-pair analysis (BreakDancer, release-0.0.1r61 [56]), and read-depth (CND [57]). Following merger of these calls into a non-redundant set, computational validation by local assembly and breakpoint refinement was performed. Details of the complete pipeline, SVMerge, are described elsewhere [28].

SV calls were intersected with the RepeatMasker [58] track of mm9 downloaded from UCSC on 20 July 2010 [59,60]. SVs were then classified as B6^+ ^TEV based on the following criteria: they must contain TE sequence as annotated by RepeatMasker, must be a deletion with respect to the C57BL6/J reference assembly and sequence annotated as TE needed to be within 50 bp of the SV breakpoints. TEVs were then further classified into superfamilies and TE structure types: LINE, fragment of a LINE, SINE, DNA transposon, LTR bound element or as more 'complex'. The LTR bound elements were further subdivided based on structure: solo-LTR (containing only a single LTR), LTR-int (the deleted sequence if comparing a provirus element to a solo-LTR), provirus (an intact ERV with two LTRs and internal sequence), pseudoelement (ERV with partial LTR on either end and/or poly-A tail), hybrid provirus (multiple RepeatMasker subfamily annotations within one repeat) or hybrid pseudoelement. A flowchart of the classification criteria can be found in Additional file 11.

Of 145,429 SVs that are absent from the C57BL/6J assembly, 11% did not appear to contain TE sequence, 21.2% TE sequences did not coincide with the SV breakpoints and 33.4% were denoted as being complex, meaning that they contained either simple repeats or multiple events of TE insertion. The categories DNA transposons (*n *= 283), LTR-int (*n *= 317), pseudoelement (*n *= 12), hybrid pseudoelement (*n *= 36) and complex (*n *= 48,521) were subsequently disregarded in further analyses. The remainder were classified into the TE repeat families corresponding to the probes used in the B6^- ^calls (Additional file 2). Although most TE families were classified based on their RepeatMasker annotations, LINE elements were classified as LINE fragments (hereafter referred to as LINE_frag) if the length of the element was less than 5 kb. The minimum cutoff was determined by the local minima between the frequency distribution curves of small fragments and the predominance of LINEs of lengths near the 6.4 kb canonical sizes (data not shown).

### B6^- ^calling algorithms

TE insertions that were present in any strain but absent from the C57BL/6J mm9 reference sequence (denoted as B6^- ^calls) were identified using RetroSeq [61]. RetroSeq seeks inconsistently mapped read pairs where one end is mapped confidently (referred to as anchor reads) but the other end is either not mapped to the reference or mapped to a distant location on the reference with low mapping quality. The non-mapping or distantly mapped mates are then aligned to the ERV probes (Additional file 2). RetroSeq requires the anchoring read to have a minimum mapping quality of 30 and at least 10 independent read pairs to support a call. Alignments to Repbase were performed with SSAHA2 [62] with a minimum of 80% identity and hit length of 36 bp. RetroSeq clusters the supporting read anchors to produce variant calls to approximately 1 to 2 kb resolution. The initial seed call windows were subject to further checking as follows. To identify the putative breakpoints, we scanned the region for positions with coverage fewer than ten reads and positions with low coverage mismatches (false alignments at the breakpoints can appear as false SNPs). For each putative breakpoint, we checked the ratio of forward/reverse orientated anchor reads at either side of the breakpoint. For the breakpoint to be accepted, we required at least 10 forward orientated anchors within 450 bp upstream and 10 reverse orientated anchors within 450 bp downstream. We also required that the ratio of forward-to-reverse anchors in the 450 bp upstream and 450 bp downstream to be less than 2-to-1. Furthermore, we required the distance from the final forward orientated upstream anchor to the first reverse orientated downstream anchor to be less than 120 bp. We then removed any calls that occurred within 50 bp of a region annotated by Repeatmasker as a 'simple_repeat' or 'low_complexity' or a SINE, LINE or ERV element in the mm9 reference.

Due to the differences in sequencing depth across the strains, it was necessary to carry out a computational genotyping step in order to correct for false negatives in the strains with lower sequencing coverage. For each TEV call and each strain that the call was not made in, we examined the reads 300 bp upstream and downstream and counted the number of putative anchor reads. If there were at least five forward orientated anchor reads upstream and five reverse orientated anchor reads downstream, then we called the TEV as being present in the strain.

### B6^- ^orientation

To determine the sense or antisense orientation of the B6^- ^elements, we carried out local *de novo *assembly with Velvet [63] of the reads that mapped within 600 bp upstream and downstream (including their mates) of the putative breakpoint. We realigned the contigs to the reference with SSAHA2 [62] and detected contigs that align incompletely at the breakpoints. We aligned the unmatched part of the contig to the ERV probe set in order to determine the orientation status of the element.

### B6^- ^size estimation

In order to obtain an accurate estimate of the sizes of the B6^- ^TEVs, we generated a single long range Illumina 'jumping' library with an estimated fragment size of 3 kb and sequenced the 50 bp of the ends of the fragments in a single HiSeq2000 lane per strain for 13 strains (129P2, 129S1/SvImJ, 129S5, A/J, BALB/cJ, C3H/HeJ, CAST/EiJ, CBA/J, DBA/2J, LP/J, NZO/SHiLtJ, PWK/PhJ, and WSB/EiJ). These data have been submitted to the European Nucleotide Archive (ENA) under Sequence Read Archive (SRA) study ID ERP000255.

Briefly, mate pair (3 kb) libraries were prepared from 10 μg mouse genomic DNA using a hybrid SOLiD/Illumina library protocol developed by L Shirley and M Quail at the Wellcome Trust Sanger Institute. Mouse genomic DNA was sheared to approx 3 kb fragments using a Digilab Hydroshear and the 2 × 50 bp mate-paired library was constructed using the nick translation protocol (*SOLiD 3 Plus System Library Preparation Guide *2009) as described elsewhere [64]. Immediately following the S1 nuclease/T7 exonuclease digest, the biotinylated mate-pair fragments were purified and ligated to appropriate adapters (Integrated DNA technologies, Leuven, Belgium), enriched by PCR then size-selected exactly as described in the *Illumina Mate-pair Library v2 Sample Preparation Guide*.

We mapped these reads to the reference genome using SMALT [65] and estimated the physical coverage from these lanes to be between 30- and 40-fold per strain. For each B6^- ^TEV in the above strains, if there were more than two read pairs spanning the insertion breakpoint, then we estimated the size of the element to be > 3 kb. This information was used to assign an approximate size status to the LINE and ERV calls. To validate this approach, we observed that almost all (> 95%) SINE calls were spanned (data not shown).

### B6^+ ^validation

An estimate of 2.6% was made for the false positive rate in the B6^+ ^calls from the percentage of TEVs from families considered to be inactive in the mouse lineage. For the B6^+ ^calls, an estimation of the true false negative rate was performed against the high confidence manually validated sets for chromosome 19 in 8 of the strains and the 250 selected PCR-validated SVs described in [26] (Additional file 4). In these PCR sets no false positives were detected.

In order to estimate false negative rates, we made a conservative assumption that the three 129-derived substrains (129P2/OlaHsd, 129S1/SvImJ and 129S5/SvEvBrd) are monomorphic for any TEV. Therefore, we counted the number of TEVs where there was a call made in two out of the three 129-derived substrains and assumed the missing call to be a false negative. For B6^+ ^calls, we obtained false negative estimates of 13.3%, 14.4%, and 9.5% for SINE, LINE, and ERV classes, respectively (Additional file 4).

### B6^- ^validation

To measure the false positive rates of the B6^- ^TEV calls, we performed 53, 34 and 47 random PCRs across SINE, LINE, and ERV superfamilies, respectively. Primers were designed using Primer3 [66] and purchased from MWG (Ebersberg, Germany). For each insertion call, several independent PCR reactions were carried out, including two reactions with Hotstar Taq (Qiagen, Hilden, Germany), and a reaction with LongRange PCR Kit (Qiagen). Reactions were performed as previously described [67]. PCR gel images were then taken to assess the performance of the PCR reaction (Additional file 6). PCR products were purified in a 96-well Millipore (Billerica, MA, USA) purification plate, resuspended in 30 μl of H_2_O and sequenced as previously described [67]. All sequencing reactions were run out on an ABI3700 sequencer and assembled using PHRED/PHRAP [68]. Consed was used for editing and visualization of the assembly [69]. Strains with and without the insertion were aligned in one single contig. Breakpoint analysis was mostly based on visual inspection of the alignment after a BLAT search. Breakpoints were identified in alignments between sequences from strains with and without the insertion. PCR results are listed in Additional file 5. From these data, we estimate false positive rates to be 22%, 11%, and 0% for the three superfamilies (SINE, LINE, ERV), respectively. From the strain distribution patterns of the PCR, we estimated the false negative rates of the calls to be 7%, 12%, 5%, for SINE, LINE and ERV, respectively. We also estimated an upper bound on the false negative rate from the 129-derived substrains to be 28%, 32% and 12%, respectively.

We compared the datasets from previous publications [18,21,23,24] to estimate the fraction recapitulated in the current TEV call sets. We capture 84% of calls from [18], and 84% of calls from [21]; 23% of the SV calls in [23] are overlapped by our TEV calls in DBA/2J; and, of the full-length intronic LINE1 elements in [24], we recapitulate 76%. The overlaps may reflect differences in filtering criteria, and the non-zero false negative and false positive rates in these and our own studies.

In a recent publication, Li *et al. *[18] compared the IAP calls and validations to the SV calls presented in [26]. The TEVs presented here have been updated since publication and so we repeated this comparison. Of 12 B6^+ ^TEVs that were considered, 3 are false negatives in our calls, which is in line with the null hypothesis that they are annotated as the C57BL6/J variant. One B6^- ^TEV appears to have been called in error in CAST/EiJ and SPRET/EiJ, which accords with expectations based on a low, but non-zero, false positive rate.

### Expression comparison methods

For 14 of the strains, RNA was extracted from the whole brain of the sequenced mouse and a female sibling at 8 weeks of age using Trizol (Invitrogen, Carlsbad, CA, USA). RNA (RNA integrity number (RIN) > 8) was then used to generate transcriptome libraries, which were sequenced on the Illumina platform. Each lane of transcriptome sequence was re-genotyped prior to downstream analysis. The raw sequencing data have been submitted to the SRA under accession number ERP000614 (links can be found at [70]).

TopHat v1.1.1 [71] was deployed to map reads passing Illumina's chastity filter from each library to the mouse genome assembly (mm9), including splice sites annotated in Ensembl and UCSC gene structures, known mRNAs, and expressed sequence tags [60], and to search for novel splice sites with a minimum isoform fraction as 0.0. Insert size and standard deviation were estimated from the full width at half maximum of the internal insert distance based on reads mapped uniquely with bwa [72]. Cufflinks v0.9.2 [73] was then used to quantify expression of all Ensembl transcripts across all libraries. Bias correction [74] and quantile normalization were both enabled, and annotated mitochondrial transcripts and ribosomal RNAs were masked when determining the denominator of the fragments per kilobase of exon per million fragments mapped (FPKM) quantification to provide maximally robust expression values.

We calculated normalized read counts for the constitutively expressed nucleotides of each gene per strain in order to produce one measure of expression level for each gene across all its transcript models. Genes with no constitutively expressed nucleotides or with no detectable expression in any of the strains were discarded. We selected 10,957 TEVs that were inserted in genes and that are variable between at least 2 of the 14 strains for which we had two biologically replicated RNA-Seq data sets. Read counts from strains with the TEV and strains without the TEV were randomly sampled and compiled into tables representing four measures (two strains each with two replicates) of expression for each gene in strains with the TEV and four for the strains without the TEV. For each gene sampled, a corresponding set was generated from genes that are without TEVs in any strain. The resulting tables were analyzed using the DESeq R package to test for differential expression between RNAseq data sets. A Benjamini-Hochberg FDR of 10% was used as a cutoff for each of 100 resampling tests. Differentially expressed genes called in at least one-third of the tests were defined as being significantly differently expressed. A schematic representation of the method is provided in Additional file 12.

### CTCF-binding TEVs and variance in expression

We intersected the B6^+ ^calls with the CTCF binding peaks provided in a recent publication [47]. Expression variance in strains with or without an intergenic TEV was estimated by taking the log natural of the ratio of expression of the immediately upstream and downstream genes where expression data were available. Orientation (that is, which was considered upstream and downstream) was randomly permuted per gene set. The null hypothesis was that there is no difference in expression variance with respect to the CTCF-binding TEV, and this was tested with analysis of variance (ANOVA) and rejected (*P *< 0.001). To test if this was a characteristic of TEVs in general, 10,000 random samples of expression ratios for TEVs not annotated as CTCF-binding were generated and tested in the same manner. The *P*-value for the CTCF-binding TEV ANOVA test was smaller than all 10,000 non- CTCF-binding TEV-associated gene expression variance samples.

### Availability of calls

The full set of TEV calls has been submitted to the Database of Genomic Variants archive DGVa at the European Bioinformatics Institute (estd118) [75] and has also been provided in BED file format (Additional file 13).

### Distribution of TEVs across a phylogeny representing a primary subspecies history of 18 mouse strains

Ignoring incomplete lineage sorting, we calculated an approximate phylogenetic tree of mouse strains that would allow us to infer the internal node whose ancestral species acquired a TEV insertion (Figure 1). Using Seqboot, Mix and Consense from the Phylip package [76], we considered the TEVs to be discrete morphologies and performed 100 bootstraps. All nodes of the resulting consensus tree were established with 100% reliability. TEVs were mapped parsimoniously to the last common ancestral node of all strains carrying the TEV.

### Structure and activities of ERV families

From the RepeatMasker [58] track of mm9 the number of bases belonging to each ERV family was calculated as the total amount of sequence annotated as one of the RepBase identifiers (Additional file 2). The proportion of ERVs that are TEVs was estimated from the number of bases in the B6^+ ^TEV calls (Figure 2b). From the B6^+ ^structure classifications of the ERV TEVs mapped to the primary phylogeny (Figure 1a) within the C57BL6/J lineage (Figure 1c), percentages of proviral-LTRs were calculated (Figure 2c, d). The average autosomal densities (TEV/bp), and the individual chromosome density ratios across all strains were also calculated (chromosome density/autosome density; Figure 3b) for the set of all TEVs. The time between nodes AB and GH was taken to be 2 My [27], which, together with the proportion of ERVs found as solo-LTRs, also allowed an approximate half-life of provirus LTR recombination rate to be calculated (λ = Y × log(1/2)/log(Z), where Y is the years of divergence and Z is the fraction of proviruses observed).

### Genome-wide nucleotide composition, gene structure and gene annotation biases for TEV occurrence

C57BL6/J genomic TEs were identified from the RepeatMasker [58] track of mm9 by excluding consecutive annotations of TEs in the same subfamily and by concatenating LTR bound sequences of a proviral structure. The local GC content was calculated from the 20 kb of sequence surrounding the TE (Figure 3a).

To calculate TEV density in different exons, introns, 5 kb flanks of genes and intergenic regions we used the Genomic Association Tester (GAT) [77]. GAT calculates an expected count through randomized simulations of the input data taking into account the observed segment length distribution. Simulations are performed per chromosome and isochore and so provide unbiased measures of the null expectation. Multiple testing corrections were applied with the Benjamini-Hochberg method [78]. GAT was also used within the various genomic spaces to test for significant association of TEVs to GOslim terms [79] (Figure 4b) and Mouse Genome Informatics overarching phenotype annotations [80-82] (Figure 4c).

### Densities of TEVs with respect to chromosome

To test the chromosomal densities of TEVs and fixed TEs, we applied a cross-genome GAT test (as opposed to the default by chromosome tests). The null hypothesis was that fixed TEs and TEVs are equally distributed across the genome when taking into account G+C biases. The null was rejected if the *P*-value passed an FDR of 0.1% within each TEV class and distribution test [78]. To account for replication timing we used data downloaded from [37,83] and classified genomic sequence as being either early or late replicating as previously applied to neutral substitution rates [84]. To take into account genic region biases, the genome was divided into genic versus intergenic space according to the release *Mus musculus *63 from Ensembl [85]. Since taking replication timing nor genic regions into account for the GAT tests did not influence the chromosomal biases, these were not included in the final analysis.

We applied the McDonald-Kreitman test [41] to chromosomal densities of TEVs and fixed TEs. Chromosomes with a higher proportion of fixed TEs compared to TEVs were significantly dissimilar as determined by a G-test based on the number of TEVs compared to the density expected from fixed TEs (FDR 0.1%) [86,87].

### Densities and orientations of TEVs with respect to the transcriptional (sense) direction of mouse genes

Orientation bias was calculated as the percentage of intronic TEVs present in the sense orientation. Dividing introns into those that are first, intervening or last within genes showed skewed distributions of TEV orientations. In Figure 5a we performed chi-square tests that showed significant differences for the occurrences of SINE and ERV TEVs depending on intronic space.

From the RepeatMasker [58] track of mm9 the sequence divergence from the prototypical sequence of each B6^+ ^TEV was taken as a measure of age of the insertion. The percent divergence was divided into bins and the trend plotted (Figure 5b).

### Densities of TEVs in the proximity of gene and exon boundaries

TEV densities were calculated upstream and downstream (Figure 6a), and in introns near exon boundaries (Figure 6b); only mouse genes with orthologs in humans were considered, since this effectively discriminates against inaccurate gene models and noncoding RNA loci. Sense and antisense orientations, relative to the direction of gene transcription, were considered separately and TEVs with unresolved orientation were disregarded in this analysis. To treat B6^+ ^and B6^- ^TEVs equivalently with respect to placing them on the reference genome (in which B6^+ ^TEVs have an identifiable length whereas B6^- ^TEVs do not), the 5' base of each TEV was taken to be the TEV's location on the reference genome, regardless of orientations for TEV or gene. This procedure assumes that TEV properties are identical on the forward and reverse strands of assembled chromosomes in the reference genome. For each genomic space, upstream, downstream or intronic sequence, the maximum distance (the half-way point to the next gene or exon) was recorded. Distance from the exon or gene was divided into bins and each scaled using the maximum number of bases in each span present in the genome. Using these scaled bin sizes, we calculated the density of TEVs (Number of TEVs within the relevant distances/Average number of bases over those distances). The bin sizes used in Figure 6a, b were taken from the Fibonacci series to allow improved visualization of the data relative to linear or logarithmic scaling.

## Abbreviations

ANOVA: analysis of variance; bp: base pair; CI: confidence interval; ERV: endogenous retrovirus; ETn: early transposon; FDR: false discovery rate; GAT: Genomic Association Tester; GO: Gene Ontology; IAP: intra-cisternal A particle; LINE: long interspersed nuclear element; LTR: long terminal repeat; MuLV: murine leukemia virus-like; My: million years; QTL: quantitative trait locus; SINE: short interspersed nuclear element; SV: structural variant; TE: transposable element; TEV: transposable element variant.

## Competing interests

The authors declare that they have no competing interests.

## Authors' contributions

KW and BY performed the SV calling from which CN performed B6^+ ^calling. TK performed the B6^- ^calling. CN, TK and BY performed the validations. WF, DA, CP, JF, TK and CN conceived and designed the study. JF performed the QTL merge analyses, and AA contributed to their interpretation. TGB performed the calling of brain RNA-Seq expression levels. CN performed analyses and statistics. CN, TK and CP wrote the core of the paper. All authors read and approved the final manuscript.

## Supplementary Material

Additional file 1 **Supplementary Table 1**. Identifiers for mice sequenced in this study. Click here for file

Additional file 2 **Supplementary Table 2**. RepBase sequences used as probes for identification of TEVs. Click here for file

Additional file 3 **Supplementary Figure 1**. Proportions of TEVs along different lineages of the phylogeny shown in Figure 1. **(a, b) **Proportions of TEV classes in the TE superfamilies and ERV subfamilies, respectively, on the 129S1/SVImJ and 129P2/OlaHsd lineages. **(c**, **d) **Proportions of TEV classes in the TE superfamilies and ERV subfamilies, respectively, on the A/J and BALB/cJ lineages. **(e**, **f) **Proportions of TEV classes in the TE superfamilies and ERV subfamilies, respectively, on the NOD/ShiLtJ and AKR/J lineages. Total numbers of predicted TEVs occurring between neighboring branch nodes are indicated below the x-axis. **(g**, **h) **Proportions of TEV classes in the TE superfamilies and ERV subfamilies, respectively, that are private to each strain. Numbers of TEVs called as being private to strains are indicated above the plots. Click here for file

Additional file 4 **Supplementary Table 3**. Summary of validation results. Percentages in parentheses denote the false negative rate estimated from concordance between 129P2/OlaHsd, 129S1/SvImJ and 129S5/SvEvBrd strains. Click here for file

Additional file 5 **Supplementary Table 4**. B6^- ^PCR true positive validation results. Click here for file

Additional file 6 **Supplementary Figure 2**. We show a representative PCR gel image for one ERV (located on chromosome 9: 98,366,615-98,366,616), one LINE (chr10:23,570,601-23,570,602), and one SINE (chr1:162,157,648-162,157,649). PCR was carried out across eight strains: A/J, AKR/J, BALB/cJ, C3H/HeJ, C57BL/6J, CBA/J, DBA/2J and LP/J. We used Hyperladder II as size marker. Click here for file

Additional file 7 **Supplementary Figure 3**. Gene annotation biases for TEV occurrence. Gene annotations that are significantly enriched (green shades) or depleted (red shades) in exons, or intronic, 5 kb flanking or intergenic regions for TEV insertions, having accounted for GC content, chromosome and lengths, and after correcting for multiple testing. **(a, b) **Gene annotations are from either the Gene Ontology (slim set) (a) or the Mouse Genome Informatics phenotypes associated with gene disruptions (b). SINE TEVs show a pattern of enrichments and depletions that is the complement of patterns observed for LINE and ERV TEVs. Click here for file

Additional file 8 **Supplementary Table 5**. Intronic TEVs associated with differential gene expression across strains. The TEVs found to be associated with differential expression against the background of all TEVs with associated expression data and equal sampling of genes with no TEV insertions. Click here for file

Additional file 9 **Supplementary Table 6**. Intronic TEVs with associated genes' brain RNA-Seq expression values. The expression differences between the associated Ensembl genes with and without TEVs are significantly different as determined by ANOVA of log expression values with an FDR < 0.05. Click here for file

Additional file 10 **Supplementary Table 7**. Intergenic TEVs with associated upstream and downstream genes' brain RNA-Seq expression values. The expression differences between the associated Ensembl genes with and without TEVs are significantly different as determined by ANOVA of log expression values with an FDR < 0.05. Click here for file

Additional file 11 **Supplementary Figure 4**. Flow chart outlining how structural variants and B6^+ ^TEV calls were classified according to various superfamily classes and whether they were full-length. Click here for file

Additional file 12 **Supplementary Figure 5**. Schematic overview of the bootstrapping sampling method used to generate the high confidence list of gene expression changes associated with TEVs. Click here for file

Additional file 13 **Supplementary file 1**. Tab deliminated file with all the TEVs with strain distribution pattern. B6^+ ^TEVs are denoted as the default '1' if the same as the reference and 'DEL' if called as absent in a strain. B6^- ^TEVs are denoted as the default '0' if the same as the reference and 'INS' if called as inserted in a strain. We conservatively estimate there to be 103,798 TEVs, although 110,930 TEVs classified in these files are due to instances of TEVs being annotated as different subfamilies in different strains (for example, LINE versus LINE_frag). Click here for file
