# Supplementary material for: The genomic landscape shaped by selection on transposable elements across 18 mouse strains
Source: Genome Biol. 2012 Jun 15;13(6):R45. doi: 10.1186/gb-2012-13-6-r45 (PMC3446317; doi:10.1186/gb-2012-13-6-r45)
Supplement: Additional file 1 — Supplementary Table 1. Identifiers for mice sequenced in this study. [file gb-2012-13-6-r45-S1.DOC]

## Supplementary Table 1: Identifiers for the mice sequenced in this study

| **Strain** | **Genome Sequence Accession Number** | **Generation of sequenced animal** | **Date of birth** |
| --- | --- | --- | --- |
| C57BL/6NJ | ERP000041 | ?+F8 | 3/1/08 |
| 129S1/SvImJ | ERP000035 | F63pF65 | 6/29/08 |
| A/J | ERP000038 | F280 | 6/6/08 |
| AKR/J | ERP000037 | F256 | 6/3/08 |
| BALB/cJ | ERP000039 | F226 | 7/30/08 |
| C3H/HeJ | ERP000040 | F258pF262 | 7/31/08 |
| CBA/J | ERP000043 | F275 | 7/27/08 |
| CAST/EiJ | ERP000042 | F90pF93 | 6/8/08 |
| DBA/2J | ERP000044 | F219pF224 | 6/10/08 |
| LP/J | ERP000045 | F195 | 6/5/08 |
| NOD/ShiLtJ | ERP000046 | F117pF121 | 6/18/08 |
| NZO/HlLtJ | ERP000047 | ?+F41 | 5/20/08 |
| PWK/PhJ | ERP000048 | F69+3+17 | 6/1/08 |
| SPRET/EiJ | ERP000049 | F78 | 2/25/08 |
| WSB/EiJ | ERP000050 | ?+F4 | 5/17/08 |
| 129P2OlaHsd | ERP000034 | Harlan7 | 8/07/08 |
| 129S5SvEv^Brd^ | ERP000036 | F17 | 18/02/09 |
