# Supplementary figures and images for: The genomic landscape shaped by selection on transposable elements across 18 mouse strains
Source: Genome Biol. 2012 Jun 15;13(6):R45. doi: 10.1186/gb-2012-13-6-r45 (PMC3446317; doi:10.1186/gb-2012-13-6-r45)

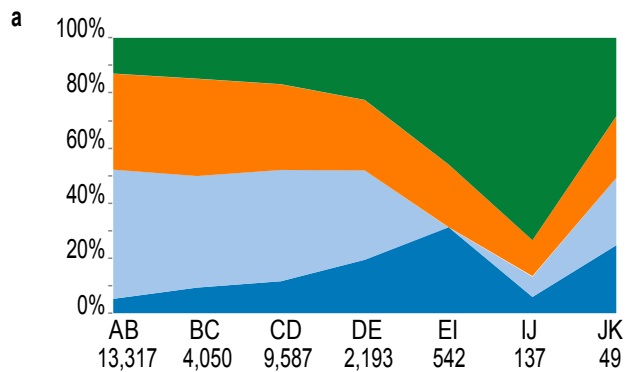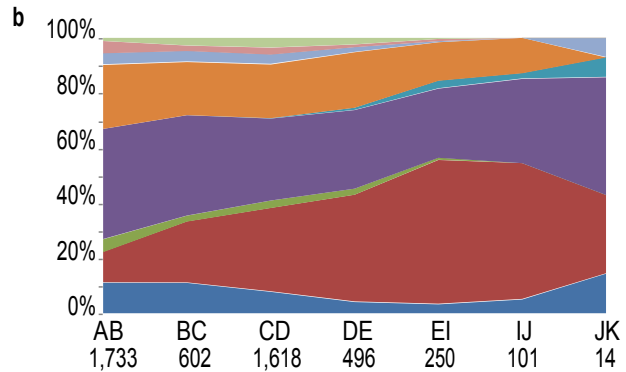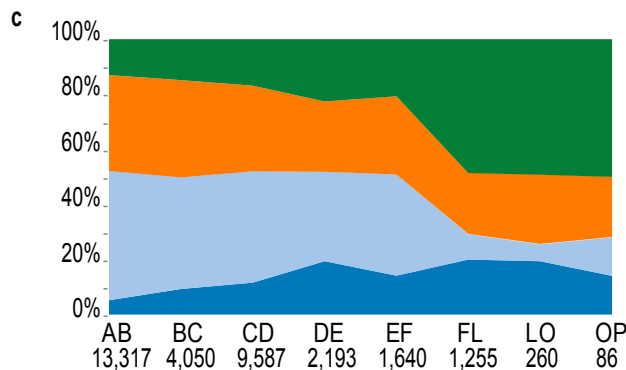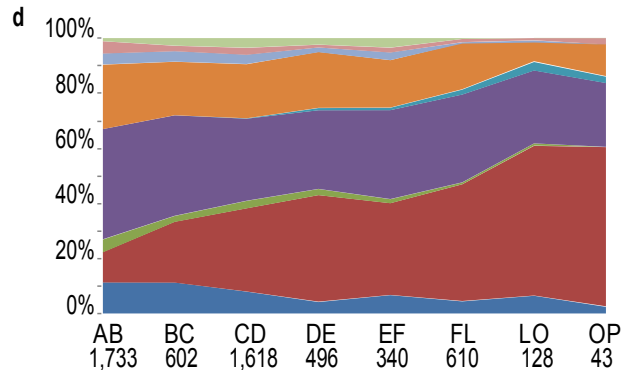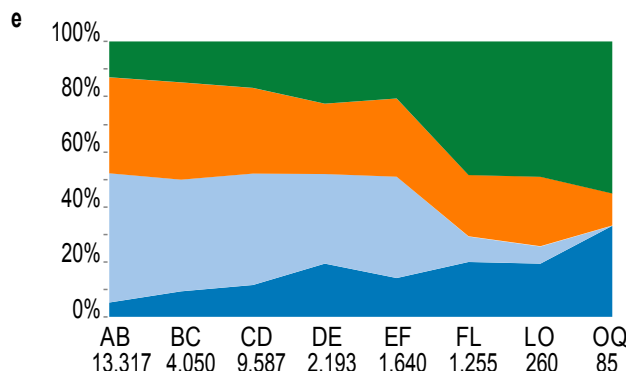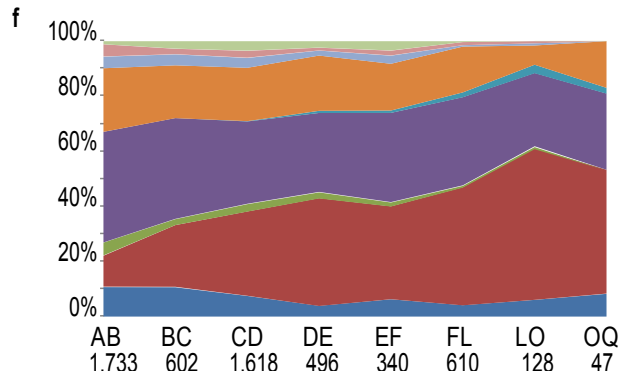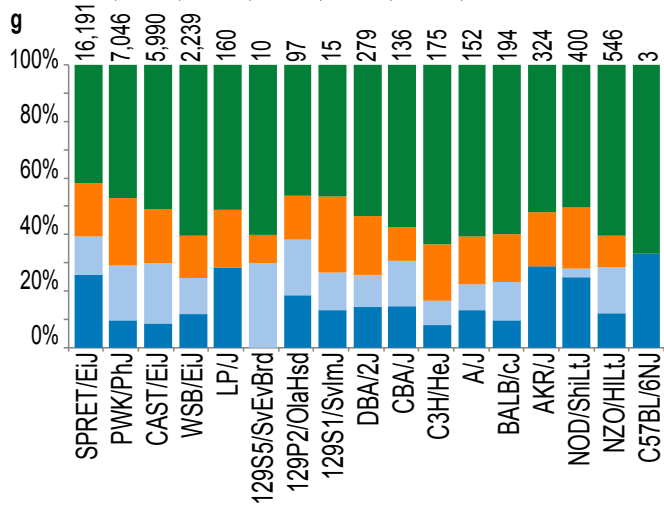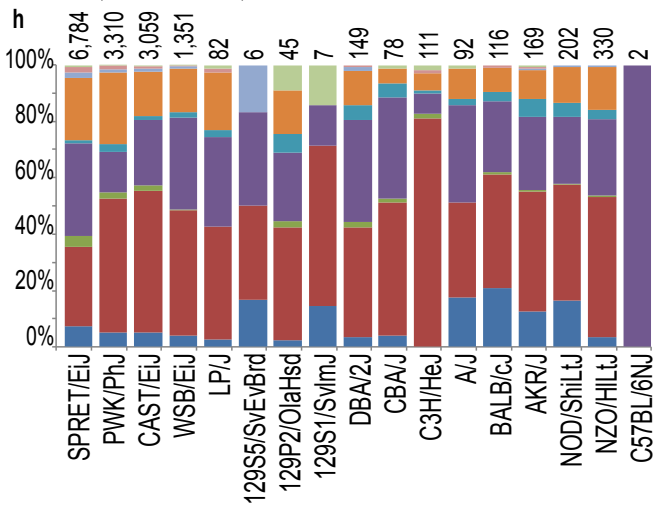

Supplement: Additional file 3 — Supplementary Figure 1. Proportions of TEVs along different lineages of the phylogeny shown in Figure 1. (a, b) Proportions of TEV classes in the TE superfamilies and ERV subfamilies, respectively, on the 129S1/SVImJ and 129P2/OlaHsd lineages. (c, d) Proportions of TEV classes in the TE superfamilies and ERV subfamilies, respectively, on the A/J and BALB/cJ lineages. (e, f) Proportions of TEV classes in the TE superfamilies and ERV subfamilies, respectively, on the NOD/ShiLtJ and AKR/J lineages. Total numbers of predicted TEVs occurring between neighboring branch nodes are indicated below the x-axis. (g, h) Proportions of TEV classes in the TE superfamilies and ERV subfamilies, respectively, that are private to each strain. Numbers of TEVs called as being private to strains are indicated above the plots. [file gb-2012-13-6-r45-S3.PDF]

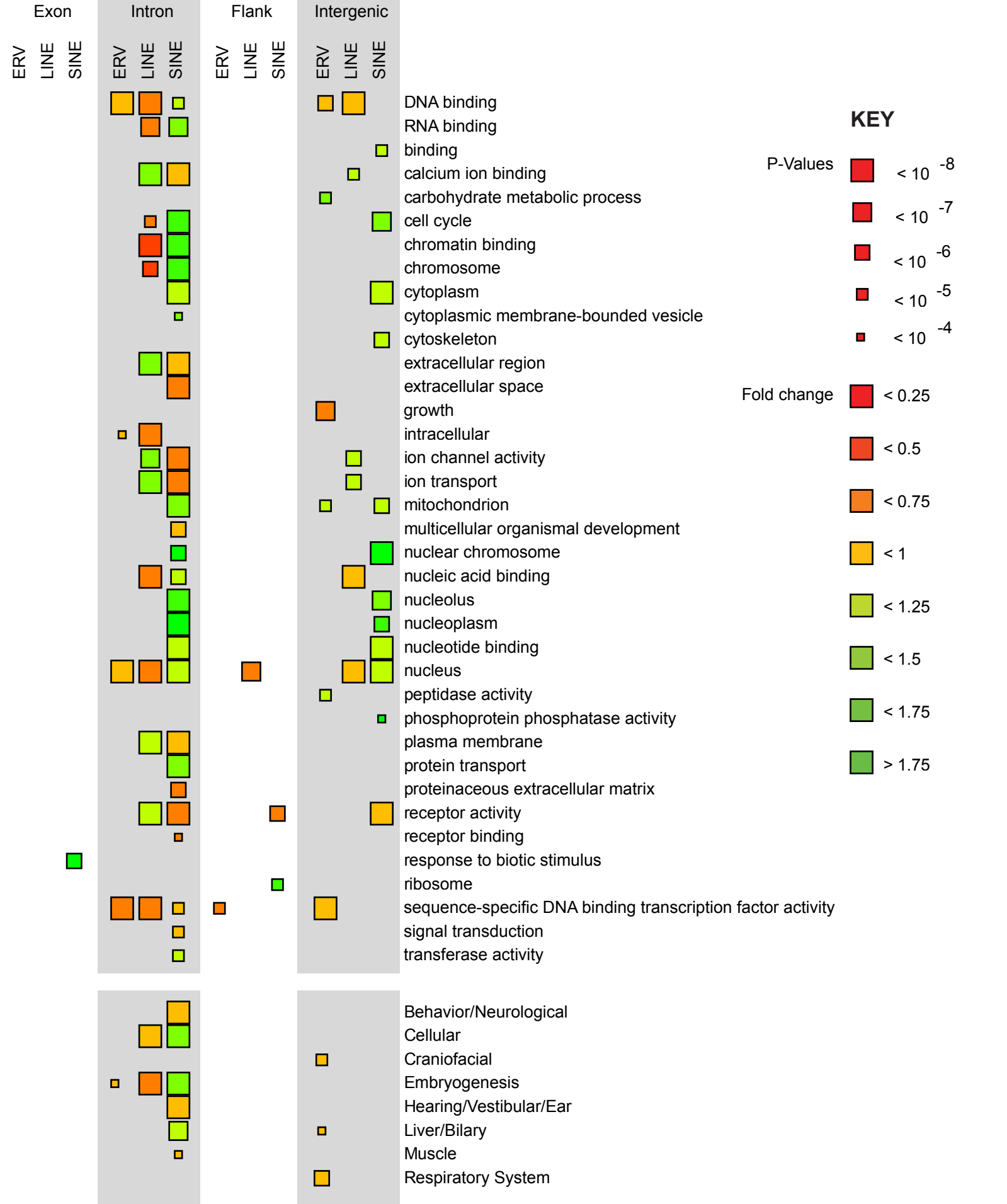

Supplement: Additional file 7 — Supplementary Figure 3. Gene annotation biases for TEV occurrence. Gene annotations that are significantly enriched (green shades) or depleted (red shades) in exons, or intronic, 5 kb flanking or intergenic regions for TEV insertions, having accounted for GC content, chromosome and lengths, and after correcting for multiple testing. (a, b) Gene annotations are from either the Gene Ontology (slim set) (a) or the Mouse Genome Informatics phenotypes associated with gene disruptions (b). SINE TEVs show a pattern of enrichments and depletions that is the complement of patterns observed for LINE and ERV TEVs. [file gb-2012-13-6-r45-S7.PDF]

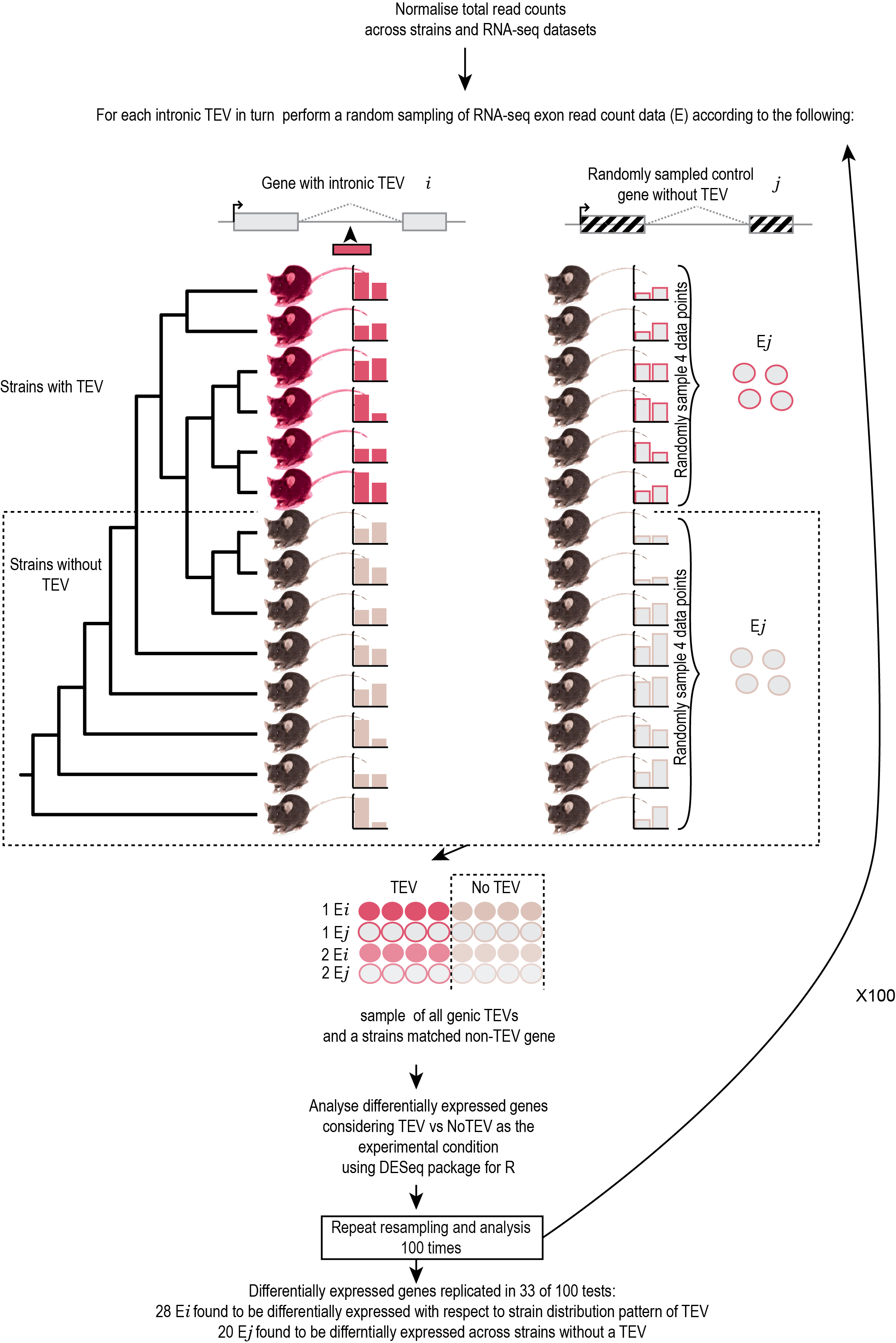

Supplement: Additional file 12 — Supplementary Figure 5. Schematic overview of the bootstrapping sampling method used to generate the high confidence list of gene expression changes associated with TEVs. [file gb-2012-13-6-r45-S12.PNG]
