# Supplementary material for: The genomic landscape shaped by selection on transposable elements across 18 mouse strains
Source: Genome Biol. 2012 Jun 15;13(6):R45. doi: 10.1186/gb-2012-13-6-r45 (PMC3446317; doi:10.1186/gb-2012-13-6-r45)
Supplement: Additional file 4 — Supplementary Table 3. Summary of validation results. Percentages in parentheses denote the false negative rate estimated from concordance between 129P2/OlaHsd, 129S1/SvImJ and 129S5/SvEvBrd strains. [file gb-2012-13-6-r45-S4.DOC]

## Supplementary Table 3: Summary of validation results

|  | B6^+^ | | B6^-^ | |
| --- | --- | --- | --- | --- |
|  | False Positive | False Negative | False Positive | False Negative |
| SINE | 0% | 17%(13.3%) | 22% | 7% (28%) |
| LINE | 0% | 12%(14.4%) | 11% | 12% (32%) |
| ERV | 0% | 13%(9.5%) | 0% | 5% (12%) |

## 
