# Supplementary material for: The genomic landscape shaped by selection on transposable elements across 18 mouse strains
Source: Genome Biol. 2012 Jun 15;13(6):R45. doi: 10.1186/gb-2012-13-6-r45 (PMC3446317; doi:10.1186/gb-2012-13-6-r45)
Supplement: Additional file 6 — Supplementary Figure 2. We show a representative PCR gel image for one ERV (located on chromosome 9: 98,366,615-98,366,616), one LINE (chr10:23,570,601-23,570,602), and one SINE (chr1:162,157,648-162,157,649). PCR was carried out across eight strains: A/J, AKR/J, BALB/cJ, C3H/HeJ, C57BL/6J, CBA/J, DBA/2J and LP/J. We used Hyperladder II as size marker. [file gb-2012-13-6-r45-S6.PPT]

## Slide 1
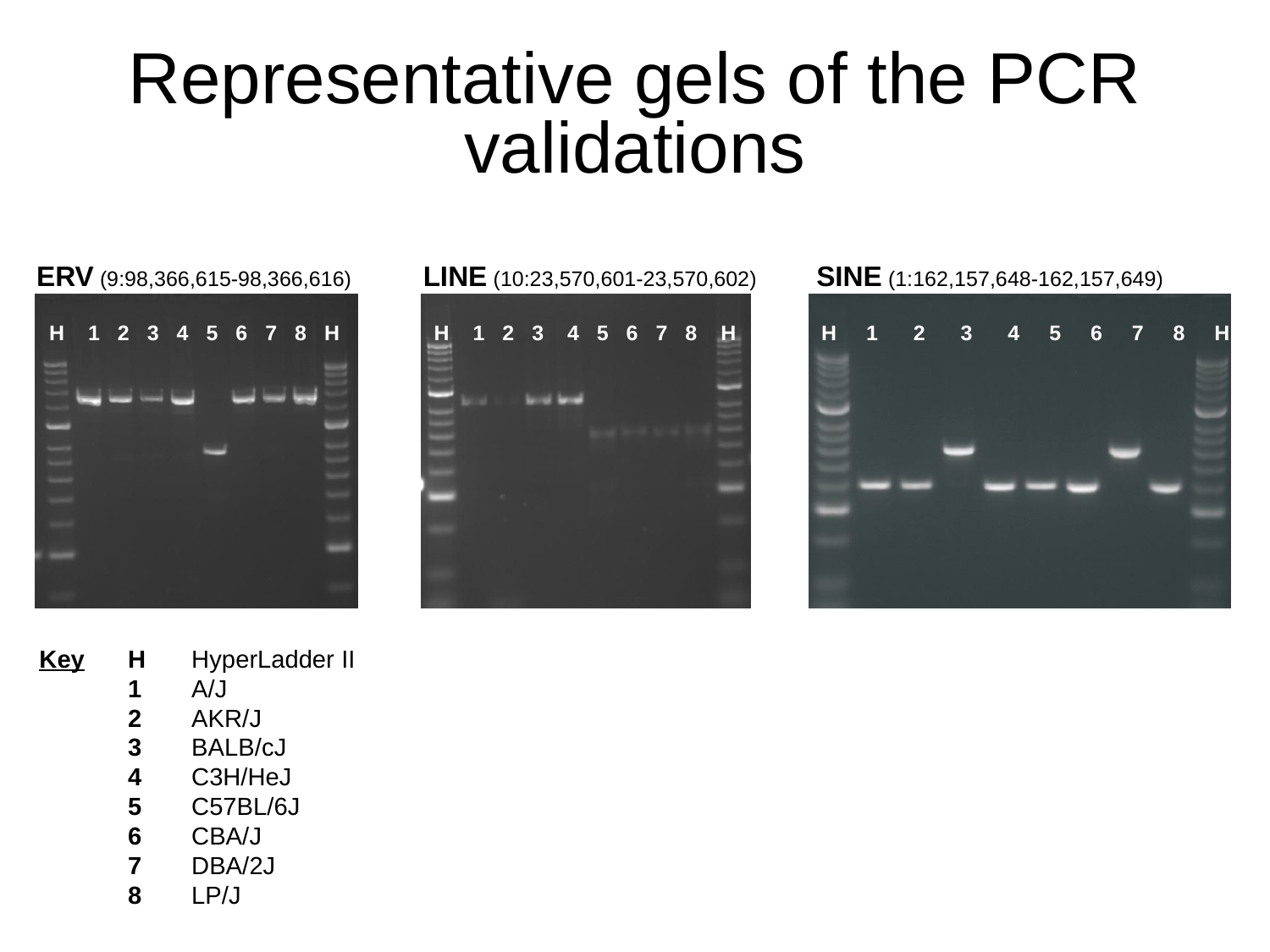

Representative gels of the PCR validations
ERV (9:98,366,615-98,366,616) LINE (10:23,570,601-23,570,602) SINE (1:162,157,648-162,157,649)
 H 1 2 3 4 5 6 7 8 H H 1 2 3 4 5 6 7 8 H H 1 2 3 4 5 6 7 8 H
H	HyperLadder II
1	A/J
2	AKR/J
3	BALB/cJ
4	C3H/HeJ
5	C57BL/6J
6	CBA/J
7	DBA/2J
8	LP/J
Key
