# Supplementary material for: The genomic landscape shaped by selection on transposable elements across 18 mouse strains
Source: Genome Biol. 2012 Jun 15;13(6):R45. doi: 10.1186/gb-2012-13-6-r45 (PMC3446317; doi:10.1186/gb-2012-13-6-r45)
Supplement: Additional file 11 — Supplementary Figure 4. Flow chart outlining how structural variants and B6+ TEV calls were classified according to various superfamily classes and whether they were full-length. [file gb-2012-13-6-r45-S11.PPT]

## Slide 1
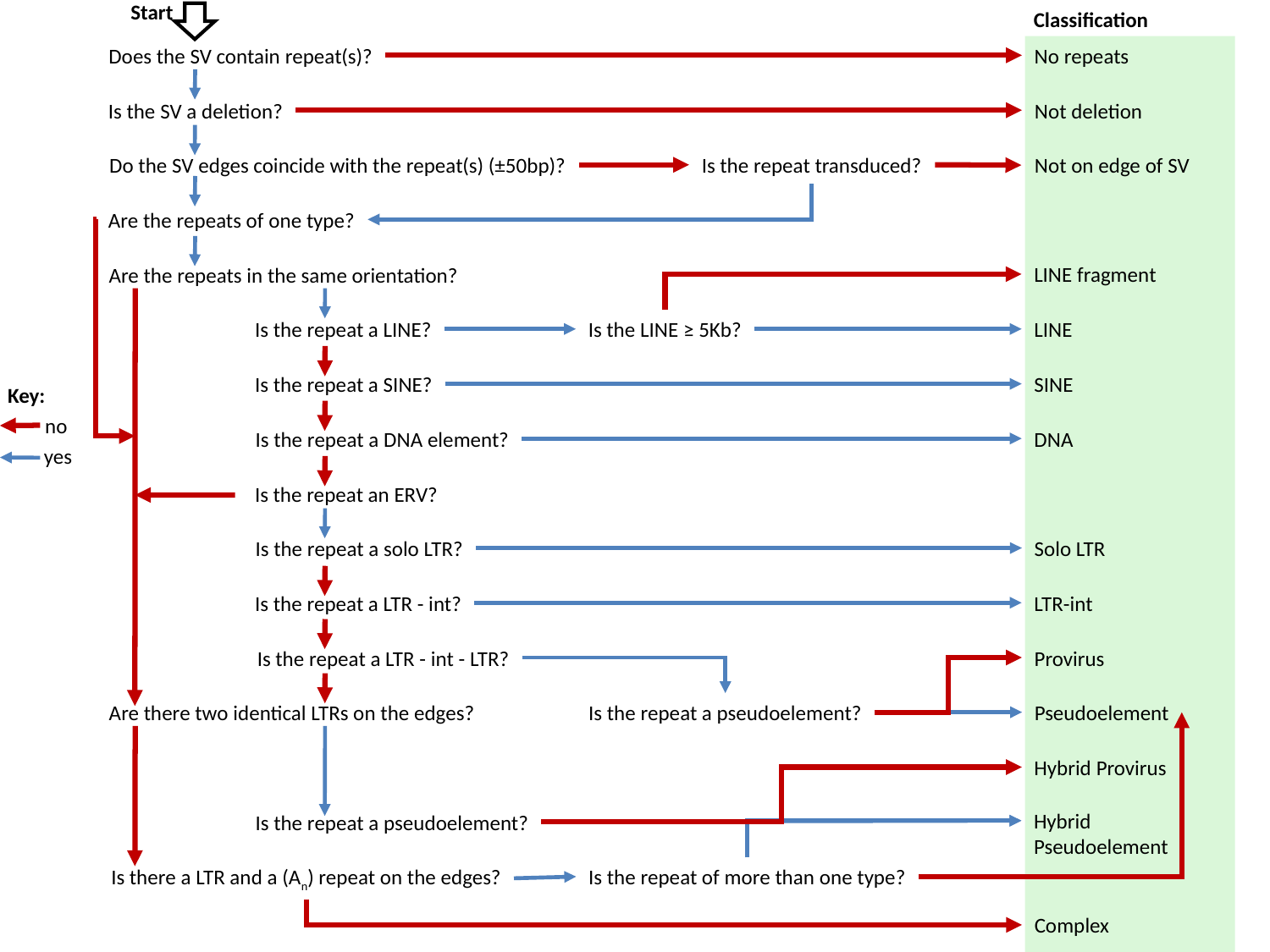

Classification
Start
Does the SV contain repeat(s)?
No repeats
Is the SV a deletion?
Not deletion
Do the SV edges coincide with the repeat(s) (±50bp)?
Is the repeat transduced?
Not on edge of SV
Are the repeats of one type?
LINE fragment
Are the repeats in the same orientation?
Is the repeat a LINE?
Is the LINE ≥ 5Kb?
LINE
Is the repeat a SINE?
SINE
Key:
no
Is the repeat a DNA element?
DNA
yes
Is the repeat an ERV?
Is the repeat a solo LTR?
Solo LTR
Is the repeat a LTR - int?
LTR-int
Is the repeat a LTR - int - LTR?
Provirus
Are there two identical LTRs on the edges?
Is the repeat a pseudoelement?
Pseudoelement
Hybrid Provirus
Hybrid Pseudoelement
Is the repeat a pseudoelement?
Is there a LTR and a (An) repeat on the edges?
Is the repeat of more than one type?
Complex
